# Cyp1b1 directs Srebp-mediated cholesterol and retinoid synthesis in perinatal liver; Association with retinoic acid activity during fetal development

**DOI:** 10.1371/journal.pone.0228436

**Published:** 2020-02-06

**Authors:** Meghan Maguire, Michele Campaigne Larsen, Chad M. Vezina, Loredana Quadro, Youn-Kyung Kim, Sherry A. Tanumihardjo, Colin R. Jefcoate

**Affiliations:** 1 Endocrinology and Reproductive Physiology Program, University of Wisconsin-Madison, Madison, WI; 2 Department of Cell and Regenerative Biology, University of Wisconsin-Madison, Madison, WI; 3 Department of Comparative Biosciences, University of Wisconsin-Madison, Madison, WI; 4 Department of Food Science and Rutgers Center for Lipid Research, Rutgers University, New Brunswick, New Jersey; 5 Department of Nutritional Sciences, University of Wisconsin-Madison, Madison, WI; Laboratoire de Biologie du Développement de Villefranche-sur-Mer, FRANCE

## Abstract

**Background:**

Cytochrome P450 1b1 (Cyp1b1) deletion and dietary retinol deficiency during pregnancy (GVAD) affect perinatal liver functions regulated by Srebp. *Cyp1b1* is not expressed in perinatal liver but appears in the E9.5 embryo, close to sites of retinoic acid (RA) signaling.

**Hypothesis:**

Parallel effects of Cyp1b1 and retinol on postnatal Srebp derive from effects in the developing liver or systemic signaling.

**Approach:**

Cluster postnatal increases in hepatic genes in relation to effects of GVAD or Cyp1b1 deletion. Sort expression changes in relation to genes regulated by Srebp1 and Srebp2.Test these treatments on embryos at E9.5, examining changes at the site of liver initiation. Use in situ hybridization to resolve effects on mRNA distributions of *Aldh1a2* and *Cyp26a1* (RA homeostasis); *Hoxb1* and *Pax6* (RA targets). Assess mice lacking *Lrat* and *Rbp4* (DKO mice) that severely limits retinol supply to embryos.

**Results:**

At birth, GVAD and Cyp1b1 deletion stimulate gene markers of hepatic stellate cell (HSC) activation but also suppress *Hamp*. These treatments then selectively prevent the postnatal onset of genes that synthesize cholesterol (*Hmgcr*, *Sqle*) and fatty acids (*Fasn*, *Scd1*), but also direct cholesterol transport (*Ldlr*, *Pcsk9*, *Stard4*) and retinoid synthesis (*Aldh1a1*, *Rdh11*). Extensive support by Cyp1b1 is implicated, but with distinct GVAD interventions for Srebp1 and Srebp2. At E9.5, *Cyp1b1* is expressed in the septum transversum mesenchyme (STM) with β-carotene oxygenase (*Bco1*) that generates retinaldehyde. STM provides progenitors for the HSC and supports liver expansion. GVAD and Cyp1b1-/- do not affect RA-dependent *Hoxb1* and *Pax6*. In DKO embryos, RA-dependent *Cyp26a1* is lost but *Hoxb1* is sustained with *Cyp1b1* at multiple sites.

**Conclusion:**

Cyp1b1-/- suppresses genes supported by Srebp. GVAD effects distinguish Srebp1 and Srebp2 mediation. Srebp regulation overlaps appreciably in cholesterol and retinoid homeostasis. Bco1/Cyp1b1 partnership in the STM may contribute to this later liver regulation.

## 1. Introduction

Cytochrome P450 1b1 (Cyp1b1) has a major influence on obesity and genes that have been linked to Type 2 diabetes [1–3]. Such changes may be initiated during pregnancy [4, 5]. *Cyp1b1* exhibits diverse expression in mouse embryo mesenchymal progenitor cells [6–8] and vascular cells [9, 10]. Expression is high in the E9.5 embryo, at sites associated with retinoic acid (RA) activity, including in the foregut region, where liver and pancreas first develop [11, 12]. Transfection of mouse *Cyp1b1* into the neural tube at this stage reproduces site- and time-specific effects associated with RA activity [13].

A functional link between Cyp1b1 and dietary retinol has been discovered from shared perinatal effects on hepatic gene regulation provided by gestational vitamin A deficiency (GVAD) and Cyp1b1 deletion. Each deliver coordinated, but distinct suppression of genes in two pathways that convert acetate to, respectively, oleoyl-CoA and cholesterol [4, 5]. One possibility is that these effects arise through earlier events in liver development. Cyp1b1/RA crosstalk at birth precedes these lipogenic effects, as shown by effects on *Hamp* and collagen genes [5]. We employ a three-step developmental model to examine effects of Cyp1b1 and retinol on postnatal liver lipogenesis. Importantly, we show that *Rdh11* and *Aldh1a1*, genes that appreciably determine liver retinaldehyde fate, fit within this network.

Cyp1b1 is absent from mouse hepatocytes, but has low expression in liver stellate cells, which store retinyl esters [14–16], and in adipose progenitors [17, 18]. This flexibility of expression is explained by a core promoter that depends on dual Sp1 complexes and several multi-element enhancers that respond to developmental regulators, such as Ah receptor (AhR), SF1 and Pax6 [19]. In mesenchymal progenitors, Cyp1b1 is induced during adipogenesis, in parallel with PPARγ [18].

Cyp1b1 metabolizes diverse natural substrates in different cell types, including estradiol in vascular cells [20, 21], arachidonic acid in macrophage [22] and natural ligands for the AhR [23, 24]. Cyp1b1 also functions to suppress H_2_O_2_ signaling in vascular and mesenchymal cells [25]. In vitro, Cyp1b1 slowly converts retinol to retinaldehyde and then to RA, but evidence for such activity in vivo is based on gene responses rather than chemical analysis [13, 26]. Cyp1b1 deletion changes gene expression in the liver in ways that mimic estradiol activation effects on growth hormone and leptin signaling [1, 2]. This signaling is realized in the hypothalamus at birth [20, 21].

The studies presented here focus on when and where Cyp1b1 expression starts to impact liver regulation. The extensive Cyp1b1 expression at E9.5 parallels early development of both brain and liver [27, 28]. Deficits in Cyp1b1 disrupt eye development in humans [29], mice [30] and zebrafish [31], but retinoid metabolism is not the source. The diversity of substrates provides many options for Cyp1b1 participation both in embryos and in the perinatal period.

During mouse embryogenesis at E9.5, the placenta delivers abundant retinol and retinyl esters, from the stores in the maternal liver [32]. However, the GVAD protocol has only a small effect on embryo levels at E14.5 [33]. Transfer of maternal retinoids begins to surge at approximately E7.5, before declining after E11.5 [34]. The expression of *Cyp1b1* in r4 of the hindbrain is second only to *Hoxb1*, a transcription factor that mediates hindbrain development [35] but remains unexplained. Hoxb1 regulates heart development [36] and is a potential regulator of adjacent liver budding through the foregut endoderm. These cells transform into hepatoblasts, which migrate into the supporting septum transversum mesenchyme (STM) [11, 12, 37, 38]. Here, we show that *Cyp1b1* is located in the STM, co-expressed with *Bco1*, an oxygenase that delivers retinaldehyde from β-carotene [39, 40]. Liver budding has been resolved into anterior stimulation of hepatoblasts by Fgf forms, and posterior stimulation by Bmp4 from the STM [41, 42].

In the mouse embryo, vitamin A/retinol from the diet delivers RA through a two-step process, involving a reversible alcohol dehydrogenase step (Rdh10 and Dhrs9) that is followed by an aldehyde dehydrogenase step (Aldh1a 1–3) [43–45]. The multiple forms of Rdh, classic alcohol dehydrogenases, and Aldh1a, evidently contribute selectively to RA synthesis according to their respective location [46–49]. At E9.5, in the region of the developing hindbrain, *Aldh1a2* establishes a gradient of RA anterior to r8 due to metabolism by Cyp26 forms. *Cyp1b1* and *Hoxb1* are co-expressed exclusively in r4, at the end of the RA gradient. Only double deletion of *Cyp26a1* and *Cyp26c1* affects this gradient, thus indicating their strong cooperation [50]. Deletions in *Rdh10* and *Aldh1a2* produce extensive losses in posterior RARE-lacZ activity [51] and shifts in rhombomere positioning [52, 53]. Additional *Cyp1b1* deletion does not add to the effects of this *Rdh10* deletion on RARE-lacZ [44].

We have looked for fetal effects of Cyp1b1 deletion and GVAD by first examining the expression of RA-regulated *Hoxb1* expression in E9.5 embryos. We have also enhanced retinoid deficiency by means of the double deletion of retinol binding protein (*Rbp4*) and lecithin:retinol acyltransferase (*Lrat*) (Lrat-/-Rbp-/-; DKO). Delivery of retinol to the embryo is compromised, particularly in combination with the GVAD protocol [33]. Malformations that are evident in combination with GVAD appear in the head at E14.5 [33] and are similar to those in Rdh10-/- embryos [52, 53]. Here, we examine these DKO embryos for the first time at E9.5. We examine how this depletion of retinol affects *Cyp26a1* and *Hoxb1* expression, as measures of local functional RA, in relation to expression of *Cyp1b1*, as a potential contributor to either RA synthesis or activity.

*Cyp1b1*, *Hoxb1*, and *Aldh1a2* also appear in close proximity in the foregut. In the perinatal mouse liver, Rdh11 and Aldh1a1 replace Rdh10 and Aldh1a2 as the most highly expressed genes metabolizing, respectively, retinol and retinaldehyde. The potential link between the steps in liver initiation and the changes in stellate cells 8 days later are of particular interest. The co-expression of *Cyp1b1* with *Bco1* in the STM provides a connection with effects of GVAD and Cyp1b1 deletion in stellate cells at birth, which derive from the STM. These parallel effects on stellate cells provide a potential developmental link to neonatal transcription changes in *Rdh11* and *Aldh1a1*. These genes function in the liver in retinaldehyde homeostasis in place of *Rdh10* and *Aldh1a2* in the E9.5 embryo.

## 2. Materials and methods

### Animal ethics statement

This study was carried out in strict accordance with the recommendations in the Guide for the Care and Use of Laboratory Animals of the National Institutes of Health. Mice were maintained in an AAALAC-accredited University of Wisconsin animal care facility. Experimental protocols were approved by the University of Wisconsin School of Medicine and Public Health Animal Care and Use Committee (ACUC; Protocol Number: M005625). Mice were provided food and water *ad libitum* and maintained in a controlled 12-hour light/dark cycle. Pregnant dams were housed singly with paper nestlets and red igloos, in accordance with approved protocols. The approved protocols were designed to minimize animal pain and discomfort. Dams were euthanized at E9.5 with metered CO_2_ asphyxiation in accordance with ACUC-approved protocols.

### 2.1 Animal care and husbandry

Wild type (WT) C57Bl/6J (Jackson Labs, Bar Harbor, ME) and Cyp1b1-/- [4] dams were maintained on standard breeder diet (2019, Harlan Teklad, Madison, WI) prior to mating and until dietary administration. Nulliparous females aged 8–12 weeks were time mated, such that the presence of a vaginal plug was designated embryonic day (E)0.5. Pregnant dams were administered purified diets at E4.5 that comprised either a vitamin A-sufficient (TD.07291, Harlan Teklad) (Suff) or a vitamin A-deficient (TD.07655, Harlan Teklad) (GVAD) diet. The purified vitamin A-deficient diet contained 0.220 IU/g of retinyl palmitate as a vitamin A source, compared to the purified sufficient diet containing 24 IU/g and 15 IU/g retinyl palmitate as in the standard breeder chow.

The Teklad 2019 and both TD diets differ in their caloric proportions of fat/carbohydrate/protein (2019, 22/58/20; TD, 12/58/20). The content of flavonoids in these diets is very low. These diets have been described previously with respect to their different content and breeding characteristics [4, 5]. The TD Suff and GVAD diets resemble the equivalent from Research Diets (D12450, 10/60/20 ratio) in lacking the three-fold iron breeder supplement. We have referred to the TD.07655 Suff diet as LF12 [4, 5].

The stage of development was assessed for each by somite number. The somite counts were rarely outside the 20–30 somite range that is expected of E9.5 staging. However, we show a wide range of embryo sizes, even with similar somite counts (S3 Fig). The three-fold variation in size within an E 9.5 treatment group with similar somite count derives from growth differences probably resultant from variation in nutrient transfer.

Lrat-/-Rbp4-/- (DKO) embryos from homozygous matings were provided by Dr. Loredana Quadro and maintained under previously described conditions [33]. The vitamin A-sufficient DKO dams were maintained on standard breeder chow [Prolab Isopro RMH3000 5p75; 18 IU of retinyl palmitate/g of diet; manufactured by LabDiet (W. F. Fisher and Son, Inc.)]. The vitamin A-deficient dietary regimen of a purified diet with less than 0.2IU/g vitamin A (Research Diets) for the DKO dams was initiated at E0.5. The vitamin A-deficient dietary regimen of the dams is abbreviated as GVAD, whether it was initiated at E0.5 or at E.4.5. These diets are distinct from those used for WT and Cyp1b1-/- studies but are breeder diets similar to Teklad 2019. Their use with WT mice has been previously described in detail [33]. The maternal vitamin A-deficient diets were initiated 5 days prior to E9.5, the start of peak maternal retinoid transfer to the fetus [32].

Embryos were isolated at E9.5 in phosphate-buffered saline within an hour. For WT and Cyp1b1-/- mice, somite number was counted (21–29 somites) to establish the developmental stage. DKO embryos were isolated at 9.5 days post coitum, regardless of developmental age. Each embryo was individually fixed in ice-cold 4% paraformaldehyde for 24 hours at 4°C. Embryos were dehydrated in increasing concentrations of methanol and stored at -20°C in 100% methanol until use.

### 2.2 *In situ* hybridization

Whole-mount *in situ* hybridization (ISH) was performed as previously described [54]. Unless otherwise stated, at least three fetuses, representing at least three independent litters, were processed concurrently using the same reagents and incubations times, such that unbiased comparisons could be made between groups. Stained embryos were oriented on 1% agarose platforms and immersed in phosphate-buffered saline for imaging. Embryos were imaged using the same lighting, objective, and magnification. The number of embryos in each treatment group are shown in supplement **S1 Table.**

To determine the average domain size of ISH stains (average of measurements per embryo), raw images were loaded into ImageJ [55] and the stained areas determined using the relative area tool. The stained areas were normalized to the total embryo area.

### 2.3 Mouse liver retinoid content at birth

For liver analyses, weaning pups were euthanized by metered CO_2_, while birth pups were decapitated on the day of birth. Liver was collected and pooled by litter, flash frozen and stored at -80°C until use. The HPLC analyses were carried out as previously described [4]. Retinol and retinyl ester peaks were resolved on a Waters HPLC system (717 autosampler, 996 UV-Visable detector, 1525 binary pump) with a C18 reverse-phase column (C18/5 micrometer, 3.9 x 300 mm column, Milford, MA). Solvent A consisted of 92.5:7.5 (v:v) acetonitrile:water and solvent B consisted of 85:10:5 (v:v) acetonitrile:methanol:dichloroethane, each with 0.05% triethylamine as a modifier.

### 2.4 Microarray analysis of liver at birth and weaning

For weaning analyses, control samples (WT/Suff) were generated from four livers from pups of different litters. Triplicate samples obtained for each treatment group were generated from pooling mRNA from two different mice. Each of three array slides were normalized by including the reference mRNA and one mRNA from each of the other three treatment groups (WT GVAD; Cyp1b1-/- Suff and Cyp1b1-/- GVAD).

For birth studies, pups were sacrificed in the PM of the first day after birth (designated 0.5). Birth liver samples are composed of two pooled litters (WT and Cyp1b1-/-, each from Suff and GVAD maternal diets). Samples were comprised of equal concentrations of pooled biological replicates. The EDGE program was used to generate sample/WT-Suff ratios for each of these comparisons.

Frozen liver was thawed in RNAlater Ice according to manufacturer’s instructions (Ambion, Foster City, CA). Total RNA was isolated from tissue by RNeasy Mini Kit accompanied by Qiashredder columns (Qiagen, Valencia, CA) according to the manufacturer’s instructions. RNA was spectrophotometrically measured for quantity and purity by A260/A280 and A260/230 on a Nanodrop, followed by visual inspection by gel electrophoresis.

RNA analyses used the Agilent Technologies 4x44k platform. Samples were prepared according to the manufacturer’s instructions for one-color labeling, including sample preparation, hybridization, and scanning. All samples were Cy3-labeled. Data was deposited in the NCBI Gene Expression Omnibus and can be accessed through GEO Series Accession Number GSE87844.

### 2.5 Statistical analysis

In our studies of the ISH imaging in E9.5 embryos, we have examined over 250 embryos that were staged by somite number. For WT mice on Suff and GVAD diets, 7 and 9 litters were used, respectively, each producing somewhat different ranges of somite numbers, corresponding to typical variation in developmental timing centered on E9.5 (range E9 to E10). For each ISH probe and treatment, we examined at least 3 embryos selected from different dams.

Comparisons of embryo cross-sectional areas and of individual mRNA *in situ* image intensities were measured as stated above and analyzed using one-way ANOVA with a Tukey post-test to determine individual p-values, where applicable. Correlation data was analyzed by two-tailed linear regression analysis. Data were graphed using GraphPad Prism (versions 5.0 and 7.0) and are represented as mean ± SEM, n = 3–6 litters/condition.

Agilent microarray data was analyzed by the EDGE3 software using the Limma analysis, which assesses significance based on ANOVA statistics [56].

## 3. Results

### 3.1 Earlier development as a source of postnatal effects of Cyp1b1 and dietary retinol

**Fig 1:**
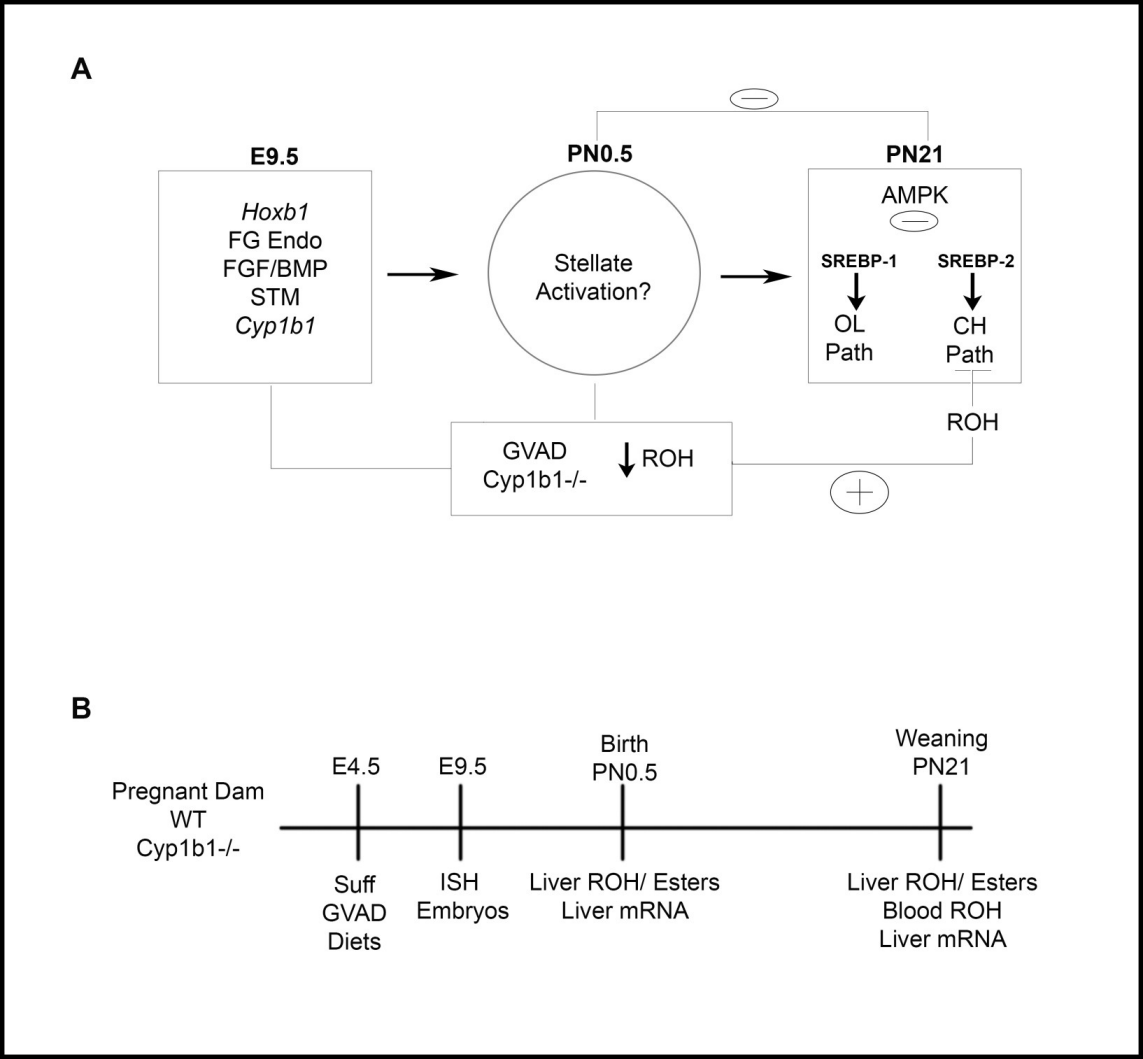
Model and design for studies of effects of Suff and GVAD diets on timed mating of WT and Cyp1b1-/- mice. Design to compare effects of GVAD and Cyp1b1 deletion at different stages of development (*Left*) Mid-gestation; E9.5 liver budding Foregut/FG, Endoderm (Endo), Septum Transversum Mesenchyme (STM). *(Center*) PN0.5; Stellate activation markers examined. (*Right*) PN21; suppression of oleate and cholesterol pathway genes (A). Design for assessment of effects of a retinol-depleted maternal diet (GVAD versus Suff) and of Cyp1b1 deletion (WT versus Cyp1b1-/- pups) on livers at birth (PN0.5) and weaning (PN21). Gene expression and retinoid content in pooled livers are compared on the day after birth (n = 3 pools) and on individual pups at PN21 (B).

The experiments shown in **Fig 1A** address a possible inter-dependence between Cyp1b1 and retinoic acid in early liver development. Lipogenic genes that convert acetate to oleoyl CoA and cholesterol surge between birth and weaning. At birth, stellate cells play important roles in liver development and storage of retinyl esters. We recently found that the postnatal increase of oleate and cholesterol pathway genes is similarly blocked by both deficiency of retinol in the maternal diet (GVAD) and in Cyp1b1-/- mice [4, 5]. This change is preceded by activation of the stellate cells by both treatments at birth (PN0.5). Here, we have tested whether these effects of Cyp1b1 deletion and dietary retinol depletion extend to retinyl esters.

This connection between stellate cells at birth and postnatal lipogenesis also raises the possibility of earlier regulatory changes in the fetus (**Fig 1B**). Thus, we have used ISH to examine GVAD and Cyp1b1 deletion in embryos at E9.5 and the effect on RA activity in the foregut at the time of liver budding. Since the liver is controlled by neuroendocrine effects from the brain, effects on *Cyp1b1* expression and genes that control RA synthesis and responses elsewhere in the embryo remain relevant.

In these studies of vitamin A/retinol deficiency (GVAD), the matching sufficient diet (LF12-Suff) differed from the standard Teklad breeder diet (lower fat/carbohydrate ratio and iron content). Litter sizes were unaffected, but body weights at weaning were approximately 10 percent smaller.

### 3.2 Retinol depletion and Cyp1b1-/- produce overlapping effects in the perinatal liver

In a previous paper, we reported that multiple genes involved in fatty acid synthesis and cholesterol biosynthesis are suppressed in Cyp1b1-/- mice at weaning and are variously sensitive to dietary retinol deficiency (GVAD) [5]. A more complete analysis shows that about 500 genes exhibit increased expression between birth and weaning (>3fold, p< 0.01), many of which function in lipogenesis. Suppression effects of GVAD at weaning are only seen with 30 genes (p<. 02), each of which is among these 500 genes that surge after birth. All of these retinol-sensitive increases are also suppressed in Cyp1b1-/- pups. The nine oleate pathway genes, therefore, represent almost a quarter of this GVAD-Cyp1b1-/- cluster. These genes are each Srebp-1c regulated. The effects for *Acss2*, the first gene in the pathway, and for *Scd1*, the terminal gene, are shown in **Table 1**. The shared suppressions in Cyp1b1-/- pups are typically larger than for GVAD maternal diet, as seen for *Acss2* and *Scd1*. There is no effect of either the diet or gene deletion at birth. Therefore, these changes are suppressing the postnatal expression surge by 85–95 percent. We have designated this response pattern as typical of Srebp-1.

**Table 1:** Shared postnatal effects on oleate and cholesterol pathways in GVAD-treated and Cyp1b1-/- pups extend to retinoic acid homeostasis.

Gene	PN21/Birth	Regulator Pattern^a^	WT GVAD/WT Suff	*Cyp1b1-/-* Suff/WT Suff	*Cyp1b1-/-* GVAD/ WT Suff
**Lipogenesis**					
*Acss2*	8.6**	Srebp1	-3.5**	-7.0**	-3.0*
*Scd1*	87.0**	Srebp1	-4.3**	-12.2**	-6.5**
*Hmgcr*	4.0**	Srebp2	NS	-6.4**	-1.5
*Sqle*	2.4*	Srebp2	-2.2*	-23.0**	NS
*Pcsk9*	7.3**	Srebp2	NS	-4.7**	NS
*Stard4*	1.6	Srebp2	NS	-2.3**	NS
*Ldlr*	2.8**	Srebp2	NS	-1.9**	NS
**Retinoid**					
*Aldh1a1*	15**	Srebp2	NS	-2.7**	NS
*Rdh11*	2.4*	Srebp1	-2.1**	-5.0**	-2.0**
*Rdh10*	0.75	none	NS	1.7*	NS
*Dhrs3*	1.5	none	NS	NS	NS

Negative sign indicates upper condition is suppressed.

**p<0.01

*p<0.05, nd-not detected, NS-response not significant.

^***a***^***Srebp-1***, pattern shown by oleate pathway genes that are mediated by *Srebp-1c*, distinguished by direct suppression by GVAD; ***Srebp2***; pattern shown by cholesterol pathway genes that are mediated by *Srebp2*, No inhibition by GVAD, reversal of *Cyp1b1-/-* effects by GVAD. The pattern does not prove involvement of these mediators for genes not previously studied.

Cyp1b1-/- pups show large suppression effects for an additional 50 genes that are scarcely affected by GVAD in the WT pups. Cholesterol pathway genes, which comprise an appreciable proportion of these genes, are each regulated by Srebp2 and show a different response pattern. The early rate limiting regulatory gene, HmgCoA Reductase (*Hmgcr*), and the late regulatory gene, squalene epoxidase (*Sqle*), are shown in Table 1. We previously showed that this Srebp2 pattern is seen for a total of twelve genes in the cholesterol pathway [4]. Typically, genes in this cluster show appreciable expression at birth, which is also insensitive to both GVAD and Cyp1b1 deletion. However, the suppressions at weaning in Cyp1b1-/- pups leaves expression at a much lower level than at birth.

Cholesterol pathway genes regain full WT expression when the GVAD diet is applied to the Cyp1b1-/- litters **(Table 1)**. This change in effect corresponds to a loss of Cyp1b1 participation when dietary retinol is low. An equivalent change is seen for the oleate pathway genes for this GVAD-Cyp1b1-/- combination. *Acss2* and *Scd1* each show an elevated expression in the GVAD-Cyp1b1-/- pups that now correspond to the suppression in the GVAD-WT pups. Thus, the participating genes lose dependence on Cyp1b1 when the diet is deficient in retinol.

In **Table 1**, we show that the dual effects of retinol and Cyp1b1 extend to *Aldh1a1* and *Rdh11*, two genes that play important roles in retinoid homeostasis [57, 58]. *Aldh1a1* shows one of the larger increases between birth and weaning but loses over 60 percent of this increase in Cyp1b1-/- pups. Like *Sqle*, this loss is completely removed when GVAD is applied to the Cyp1b1-/- dam. *Rdh11* shows an expression pattern that is very similar to that of *Acss2*.

Aldh1a1 is the major form in liver for the synthesis of RA. *Aldh1a2* and *Aldh1a3* are expressed in the embryo but were scarcely detected in these neonatal livers. Similarly, Rdh10 is the major form in the embryo but has lower expression than Rdh11 in the neonatal liver [47, 57]. In the embryo, Rdh10 also combines with Dhrs3 to function as a reductase [58]. Rdh11, however, functions alone as an NADPH retinaldehyde reductase [57, 58]. *Rdh10* is increased in Cyp1b1-/- pups, thereby becoming the major form. Dhrs3 may become functionally important by partnering with Rdh10 when Rdh11 is not the dominant form. The functional significance of Rdh11 has been shown in Rdh11-/- mice through diminished generation of retinyl esters, particularly when β–carotene is the dietary source [57].

Retinoid homeostasis has been shown to be highly integrated with cholesterol homeostasis [59]. Here, we show that this pattern of change is very specific. For example, *ACAT* forms *1 and 2* [60], which control the formation of cholesterol oleate do not show such changes. However, *Ldlr*, *Pcsk9* and *StARd4* share this pattern and have previously been linked to Srebp2 [61–63] **(Table 1)**. They each play roles in cholesterol transport. Pcsk9 is an inactivator of Ldl receptor protein [61], which delivers ApoE-cholesterol ester to the endosome network. Stard4 distributes cholesterol between different membrane sites [62, 63].

**Fig 2:**
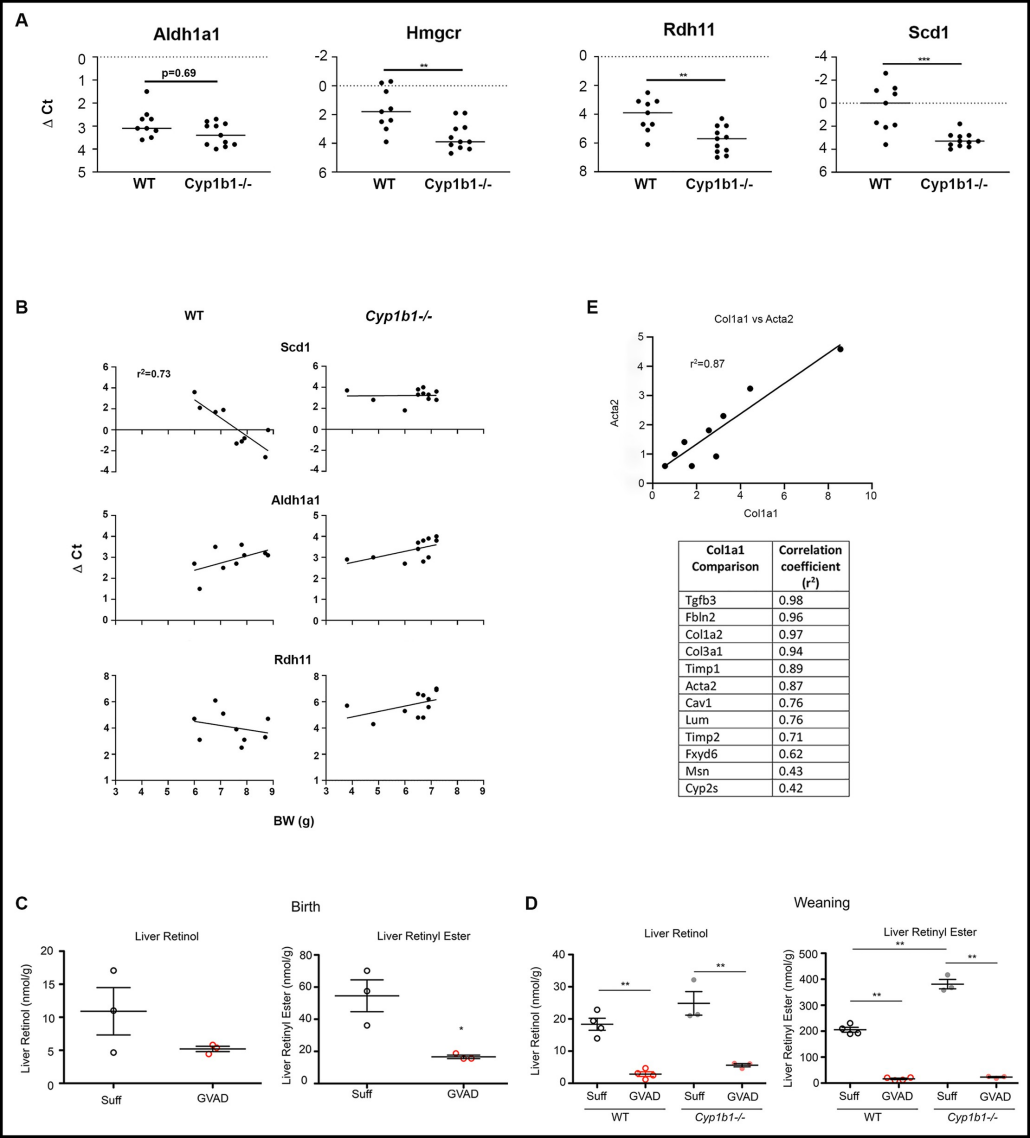
Effects of GVAD and Cyp1b1 deletion on liver retinoid content and gene expression at birth and weaning. PCR determinations of expression (dCt) for individual WT and Cyp1b1-/- mice from multiple litters at PN21: *Aldh1a1*, *Rdh11*, *Scd1*, and *Hmgcr* (A). Correlations for expression versus body weight for WT and Cyp1b1-/- mice (B). Liver retinol and retinyl ester content at birth in WT (open circles) and Cyp1b1-/- (solid circles) vitamin A-sufficient (Suff; black) and GVAD (red) mice were measured on the pooled livers from separate litters (C). Equivalent measurements on livers from individual WT and Cyp1b1-/- at weaning/PN21 pups (D). Correlation of GVAD and Cyp1b1-/- effects on stellate markers at birth (C). Correlation between *Col1a1* expression and *Sma1* and then other stellate activation markers at birth for expression in individual mice across the four treatments shown in Table 2 (E). Parts C and D were previously presented as separate supplementary figures [5]. *p-value ≤ 0.05, **p-value ≤ 0.01, ***p-value ≤ 0.001.

We used PCR to compare the expression of these genes in the same individual pups. Each treatment group was comprised of all male pups from three separate litters. In WT mice, the expression level for *Scd1* was closely correlated with body weight (BW; r^2^ = 0.73). The link for *Hmgcr* in WT mice was relatively weak (r^2^ = 0.33) (**Fig 2B**). The intra-litter variations in BW were appreciable for the three litters used for these groups. In contrast to WT pups, Cyp1b1-/- expression of *Scd1* was independent of body weight. Based on the plots shown in **Fig 2B**, there are no differences for *Scd1* expression between WT and Cyp1b1-/- for small pups (BW< 7 g). However, divergence becomes substantial as BW increases, leading to the overall significant difference shown in **Fig 2A**.

Like *Hmgcr*, the expression levels of *Aldh1a1* and *Rdh11* were independent of BW in these same WT mice (**Fig 2**). When all the WT and Cyp1b1-/- pups were compared, the differences were highly significant for *Scd1*, *Hmgcr* and *Rdh11*, despite the large BW variation for WT pups. For *Aldh1a1*, the difference indicated by the array data did not reach full significance. The array design used pooled livers for each of triplicate samples and the more powerful normalization provided by the EDGE processing algorithm.

### 3.3 Suppression of retinoids in perinatal livers by GVAD is associated with stellate cell activation, which is also sensitive to Cyp1b1

Stellate cells play an important role in liver development by providing extracellular matrix proteins [64]. In adult livers, retinyl esters are primarily stored in stellate cells, which comprise about five percent of liver cells [14]. Liver retinol and retinyl esters were extensively suppressed by GVAD at birth and weaning/PN21 (**Fig 2C and 2D)**. The liver retinyl esters are nearly four times lower at birth than at weaning and over 10 times lower than in adults [5]. Cyp1b1 deletion doubled PN21 retinyl esters (p<0.01) but with only a small effect on retinol. There was no significant difference in the liver retinol levels on the Suff diet between birth and weaning/PN21. This consistency is indicative of an effective homeostasis for liver retinol at both times, even as the stored retinyl esters increase from birth to weaning. This homeostasis is evidently disrupted at both times by the suppression effected by GVAD (**Fig 2D**). The serum retinol level is unaffected by either GVAD or Cyp1b1-/-. In adult mice, the retinyl esters are mostly stored in stellate cells.

In view of these substantial changes in liver retinol and the shared effects of the GVAD diet and Cyp1b1 deletion at weaning, we looked for equivalent effects on transcription immediately after birth. We focused on the iron sensor, *Hamp*, which shows dual effects at weaning and searched for other genes that showed dual GVAD-Cyp1b1 sensitivity. We also probed genes that are known to participate in liver retinoid homeostasis. In WT offspring, GVAD suppressed transcription of *Hamp*. Cyp1b1 deletion resulted in a still larger suppression. *Hamp* is the only gene that retains a parallel effect of GVAD and Cyp1b1-/- from birth to weaning.

GVAD produced a very limited group of fifteen stimulations that reached the two-fold threshold with significance (p<0.05). Cyp1b1-/- offspring produced a parallel set of very similar expression increases for these GVAD-responsive genes. However, overall there were substantially more stimulations in Cyp1b1-/- pups than for the GVAD treatment of WT pups. Despite the large decrease in liver retinol produced by GVAD, none of the genes typically associated with retinoid homeostasis was significantly affected (**Table 2**).

**Table 2:** Stellate cell markers are elevated in liver at birth by GVAD and Cyp1b1 deletion.

	Fold Change		Relative Expression (Cy3 x10^3^)		
*Upper vs*.	WT GVAD	*Cyp1b1-/-* Suff	Birth	Weaning^a^	Adult^a^
*Lower*	WT Suff	WT Suff			
**Hepatocyte**	**Expressed**				
*Hamp*	-2.5*	-8.8**	31.0	4.5	17.6
**Stellate**	**Expressed**				
*Acta2*	1.7 (p = .07)	2.2*	0.35	0.8	0.1
*Col1a1*	3.4**	3.9*	0.9	1.3	0.1
*Col1a2*	2.6*	4.6**	4.7	7.0	1.5
*Col3a1*	2.8*	2.5	2.9	4.2	0.5
*Fibulin2*	2.5*	2.0*	0.5	0.4	0.1
*Lumican*	2.4*	2.0*	0.6	0.5	0.4
*Caveolin1*	2.5**	2.0	0.3	0.2	0.08
*Timp1*	2.0*	1.3	0.3	0.2	0.2
*Timp2*	1.7*	1.9*	2.4	2.9	2.0
*Tgfb3*	1.8*	1.6	0.2	0.3	0.09
*Fxyd6*	2.1*	4.0	0.2	0.2	0.07
*Cyp2s1*	2.6 **	1.5	0.15	0.3	0.04
*Mesothelin*	2.6**	1.4	0.7	0.7	0.08
**Retinoid**	**Homeostasis**				
**Stellate**					
*Lrat*	-1.5 ^0.1^	-1.5 ^0.1^	0.1	0.2	0.4
*Cyp26b1*	ns	ns	0.4	0.8	1.0
*Bco1*	n.s.	n.s.	0.4	0.3	0.1
**Hepatocyte**					
*Dhrs3*	n.s	n.s	5.0	2.3	7.0
*Rdh10*	n.s	n.s	0.4	0.8	1.5
*Rdh11*	n.s	n.s	1.3	3.1	1.9
*Rdh12*	n.s.	-2.5**	2.1	1.1	0.8
*Aldh1a1*	n.s.	-2.0*	0.8	13.0	61.3
*Cyp26a1*	n.s.	1.7*	0.01	0.09	0.5
*Rbp4*	n.s.	n.s.	288	257	250
*Crbp1*	n.s	n.s	25.7	8.7	2.2
*Crbp2*	n.s	-4.5**	0.5	< .01	< .01

^a^ Weaning: pnd 21; Adult: pnd 98.

*p-value ≤ 0.05

** p-value ≤ 0.01

^01^ p = 0.1

Remarkably, the GVAD-induced increases were mostly associated with the expression of mRNA that produce matrix proteins and other genes associated with activated stellate cells. There are increases in three forms of collagen along with three other matrix proteins: fibulin, mesothelin, and Fxyd6 (**Table 2**) [14]. Other increases include the membrane regulator, caveolin 1, the oxygenase, Cyp2s1, smooth muscle actin (Sma/Acta2), Timp1, Timp2 and Tgfb3. Application of the GVAD treatment to Cyp1b1-/- had no further effect on elevated expression of these genes (not shown). This study provided ten samples of liver mRNA, corresponding to four treatment groups, with each sample corresponding to livers pooled from pups comprising a separate litter. The respective gene expression levels, based on array binding (Cy3) for the individual samples, were correlated to expression levels for *Col1a1* for the same samples. The closest match to *Col1a1* is seen for *Acta2* (r^2^ = 0.87), the classic marker for stellate cells. The pairwise correlations with *Col1a1* are shown in the **Table accompanying Fig 2E**. All but Cyp2s1 and Msn appreciably exceed the correlation between *Col1a1* stimulation and *Hamp* suppression (r^2^ = 0.5).

The basal expression for most of these stellate activation markers at birth and weaning is 3–5 times higher than in adult livers (**Table 2**). By contrast, the expression of *Lrat* and *Cyp26b1*, which are each involved in retinol and RA homeostasis in stellate cells, are lower at birth. This trend extends to genes that control retinoid homeostasis in hepatocytes. Thus, *Aldh1a1* and *Cyp26a1*, which, respectively, generate and metabolize RA, are 50 times lower at birth than in the adult, with weaning levels intermediate. Aldh forms 1a2 and 1a3, which sustain RA in the embryo, have very low expression in the liver. However, the major intracellular retinol transfer protein, CRBP1 (RBP1), is highest at birth. Rbp4, which sequesters retinol in the blood, remains constant at very high levels of expression, consistent with the maintenance of constant serum retinol levels.

This shift in gene expression from transfer to synthesis is consistent with the maternal diet and liver functioning as the near exclusive source of retinoids for the fetus. Homeostasis in the face of diet changes provided by the GVAD protocol is determined by the maternal liver. In addition, the changes effected in the livers of Cyp1b1-/- progeny are small compared to changes conferred by development. Other retinoids and pathways may be more important at birth. Thus, Bco1, which generates retinaldehyde from β-carotene, locates to stellate cells and is higher at birth [39, 40, 57]. Other potential participants may be more important at birth, including RXR ligands. The retinol saturase (Resat) and the phosphatidyl choline transfer protein (Pctp/Stard2) are each highly expressed at birth and decline significantly by with GVAD [65–67].

Stellate cells release signaling proteins that participate in injury repair [64] and may play an important role in the liver adaptation at birth. In the livers at birth, the low expression of genes associated with retinoid synthesis and the low levels of retinyl ester storage contrast with the high expression of proteins associated with retinol binding.

Crbp1 is also expressed in stellate cells but hepatocyte expression predominates in liver. The large increases in stellate activation markers point to an appreciable change in the regulation of stellate cells at birth. These effects of GVAD on the liver may be directed locally within the liver but this is unlikely for Cyp1b1, which is scarcely detectable in liver. We have looked for potential sources of this partnership at E9.5, which corresponds to a time very soon after the initiation of liver budding.

### 3.4 The impact of the GVAD diet and Cyp1b1 deletion on embryo development at E9.5

**Fig 3:**
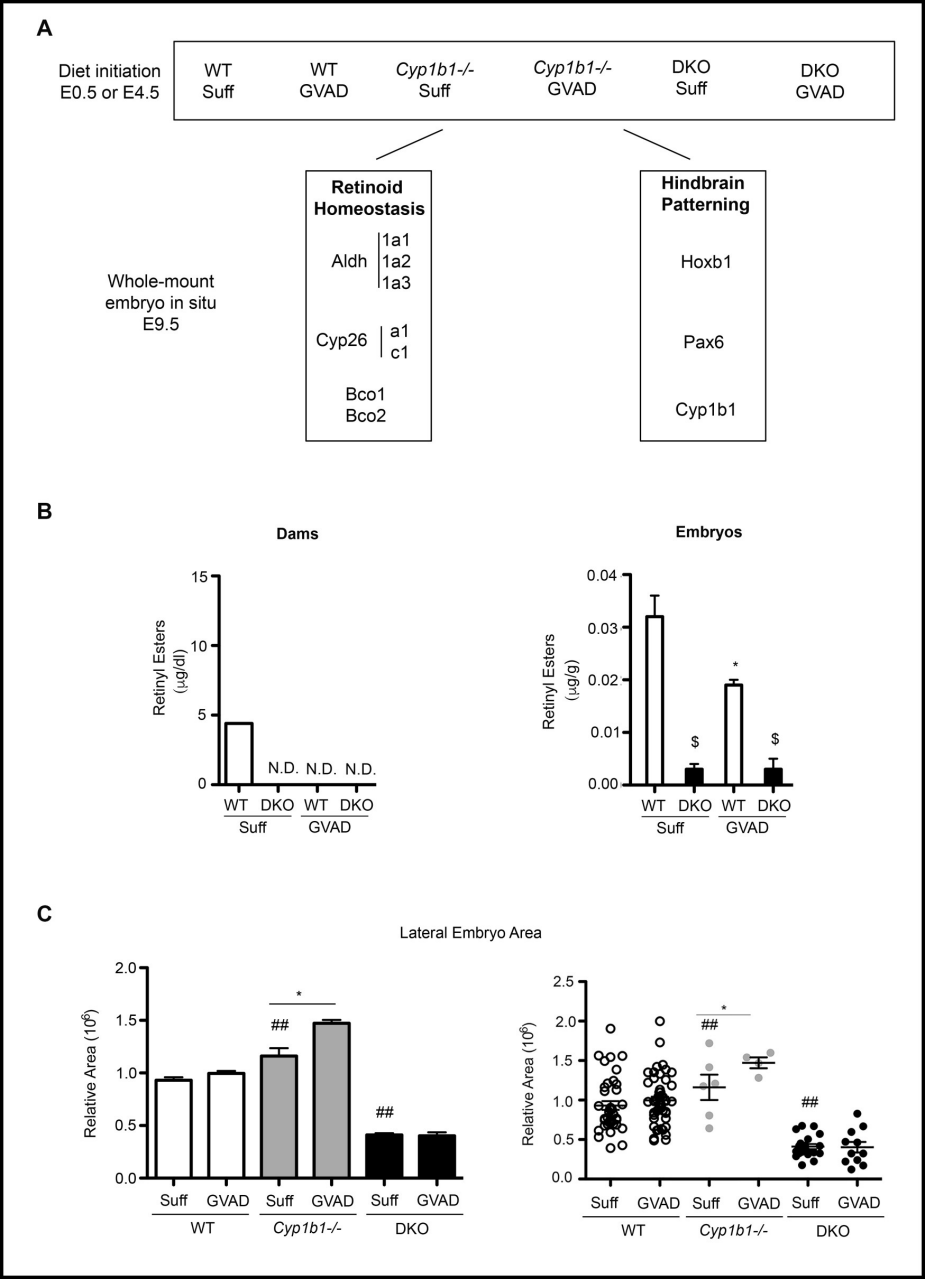
Retinoid homeostasis in WT, Cyp1b1-/- and DKO mice. Experimental design for embryos at E9.5 and pups at birth and weaning. C57Bl/6J (WT), Cyp1b1-/-, and DKO pregnant dams were administered vitamin A-sufficient (Suff) or -deficient (GVAD) diets at E4.5 (or E0.5 for DKO dams). Whole mount *in situ* hybridization (ISH) at E9.5 was used to observe the intensity and location of genes involved in retinoid homeostasis and developmental patterning (A). Depletion of serum retinol and retinyl esters at E14.5 in DKO dams and fetuses on Suff and GVAD maternal diets. Data previously published as a Table [33] (B). *p-value ≤ 0.05 compared to sufficient within the same genotype. $p-value ≤ 0.05 compared to WT on the same diet. N.D., Not detectable (<0.01 μg/dl). E9.5 embryo sizes obtained from dorsal projections for the six treatment groups (C).

To test whether GVAD and Cyp1b1 deletion produce shared effects earlier in development, we examined embryos at E9.5 with the same four treatment groups (**Fig 3A**). E9.5 represents a time when retinoid transfer from dam to embryos is at a peak [32]. After this surge, at E14.5, the GVAD diet completely removes stored retinyl esters from the liver of the dam, but only halves levels in the embryo (**Fig 3B**) [33].

At E14.5, serum retinol was only decreased by 40 percent in the dam and not in the embryo. In later experiments, we examine embryos carrying a double deletion of *Lrat* and *Rbp4* (DKO) that disrupts this homeostasis [33] (**Fig 3A**). DKO dams show a halving of serum retinol at E14.5 when using a Suff diet, which, however, does not extend to the embryos (**Fig 3B**).

GVAD and Cyp1b1 deletion have no effect on survival to weaning (PN21, 6–7 pups/litter), on body weight [5] or on the male to female ratio (data not shown). The stage of development was rarely outside the 20–30 somite range expected of E9.5 staging (Suff mean was 23 somites; GVAD mean was 25 somites). Occasional outliers were not further examined (34 pregnancies, 36/250 embryos). Somite numbers from embryos of a single dam were closely clustered, varying more between litters.

The sizes of the WT embryos showed a three-fold range for both Suff and GVAD diets, despite the narrow somite range (**Fig 3C**). Somewhat higher somite numbers were prevalent with larger embryos, but appreciable size differences were seen with similar somite numbers, especially in comparisons between litters. Growth differences can separate from development stage to affect size. DKO embryos at E9.5 also showed a three-fold size range that was appreciably lower than for WT embryos. The ISH imaging with staging markers, such as *Pax6* and *Hoxb1*, show that DKO embryos are smaller on average that WT embryos, even when reaching the full E9.5 stage of development. Delayed development was also seen as a contributor to lower body weight. The GVAD DKO embryos produced fewer embryos per dam because of enhanced resorption [33]. There were also more malformed embryos that were not assessed for size.

### 3.5 Location of *Cyp1b1* at E9.5 in relation to retinoid producing genes

The whole mount ISH probing of the mRNA expression compares *Cyp1b1* patterning to the distributions of mRNA from 8 genes that are directly associated with RA homeostasis, either through synthesis (three *Aldh1a* forms and *Bco1*) or metabolism (two *Cyp26* forms). To gauge local RA responses, we examined *Hoxb1*, which is remarkably sensitive to RA in hindbrain r4 and foregut, along with *Pax6*, which provides a constraint on RA activity [68]. In **S1 Table**, we show the number of embryos that were staged and imaged by ISH. The WT Suff and GVAD were taken from, respectively, 7 and 9 dams.

**Fig 4:**
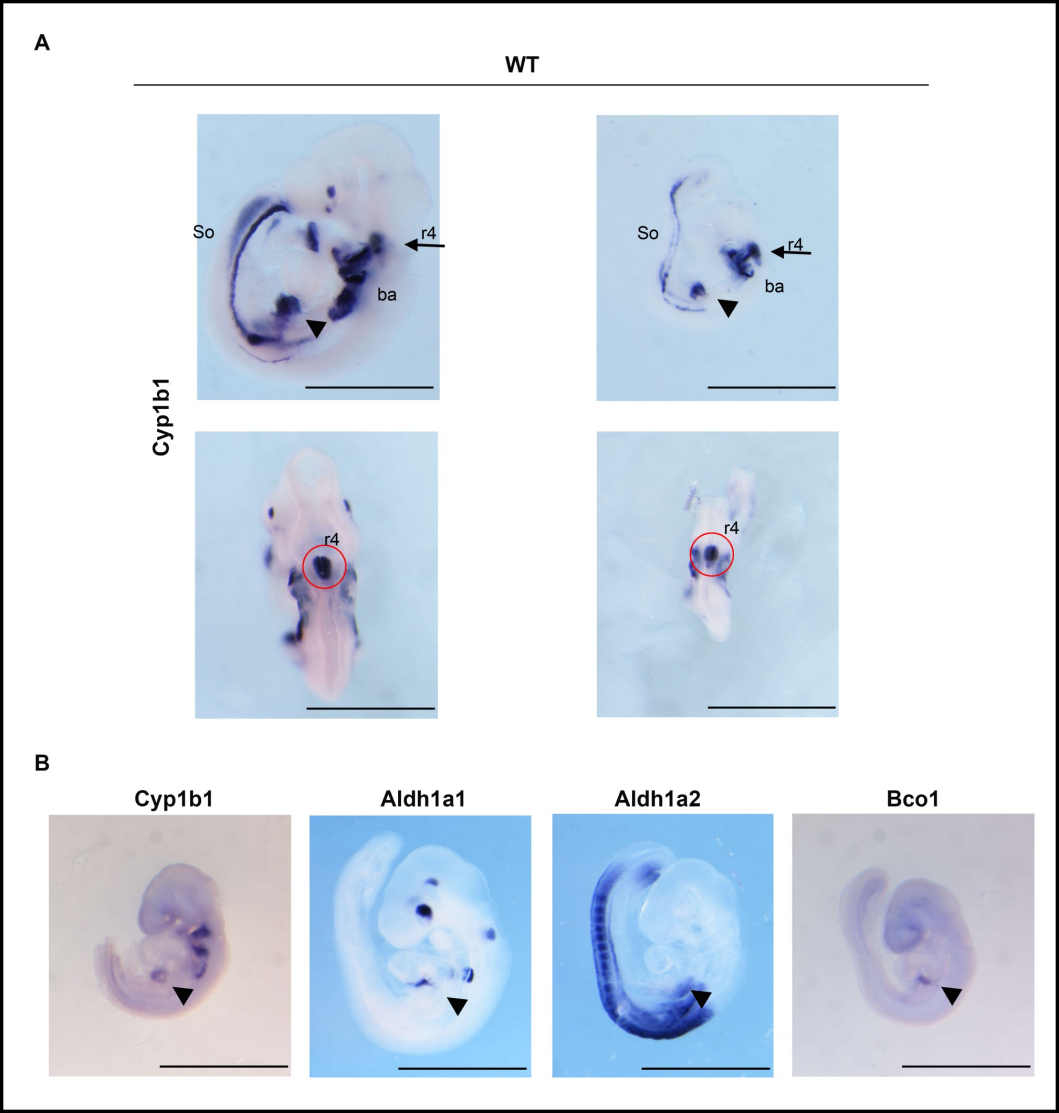
*Cyp1b1* expression in the embryo at E9.5; overlap with *Bco1* in the STM. E9.5 WT embryos of different sizes from dams administered a vitamin A-sufficient (Suff) diet were probed for *Cyp1b1*. Lateral (upper) and dorsal (lower) views are shown for two stained embryos. Noted are hindbrain rhombomere 4 (r4, ventral side), branchial arches 2, 3, and 4 (ba), septum transverse mesenchyme (STM) (arrow head), and tail somites (So) (A). *Cyp1b1* is adjacent to *Aldh1a2* and *Aldh1a1* in the foregut (arrow head) and r4 (red circle) and overlaps *Bco1* in the STM (arrow head) (B).

**Fig 4A** shows the distribution of *Cyp1b1* mRNA at E9.5 at multiple sites. Here, two embryos differ in dorsal area by roughly three-fold yet retain a similar distribution of *Cyp1b1* between the different expression sites. *Cyp1b1* is highly expressed, specifically in r4 neural crest cells of the hindbrain and in the three branchial arches. These structures derive from cells that migrate from specific rhombomeres of the neural crest (r4 to Ba2; r5 to Ba3; r7 to Ba4). The third major site of expression is the foregut, more specifically the STM, which participates in liver bud expansion [42]. *Cyp1b1* mRNA is present in caudal somites, notably with clear left/right polarization. There is also localized expression of *Cyp1b1* in the eye, which functions independent of RA [31].

*Cyp1b1* expression in the hindbrain, branchial arches, foregut and somites is mostly linked to specific sites of RA synthesis derived from *Aldh1a2* expression (**Fig 4B**). *Aldh1a1* is positioned upstream of *Cyp1b1* (at r6/r7) in the RA hindbrain gradient and in the foregut/STM region [69]. The r4 and r6/r7 provide sites from which neural crest cells migrate to the branchial arches 2–4. *Aldh1a2* is expressed more extensively than *Cyp1b1* in the somites and appears at foregut sites. *Bco1*, an oxygenase, which generates retinaldehyde from β-carotene is expressed close to *Cyp1b1* in the STM [40]. Thus, if *Cyp1b1* participates in RA activity, *Aldh1a1*, *Aldh1a2* and *Bco1* are well positioned as partners.

### 3.6 Spatial resolution of *Cyp1b1*, *Cyp26c1*, *Hoxb1*, and *Pax6* in the foregut in relation to hindbrain and branchial arches

**Fig 5:**
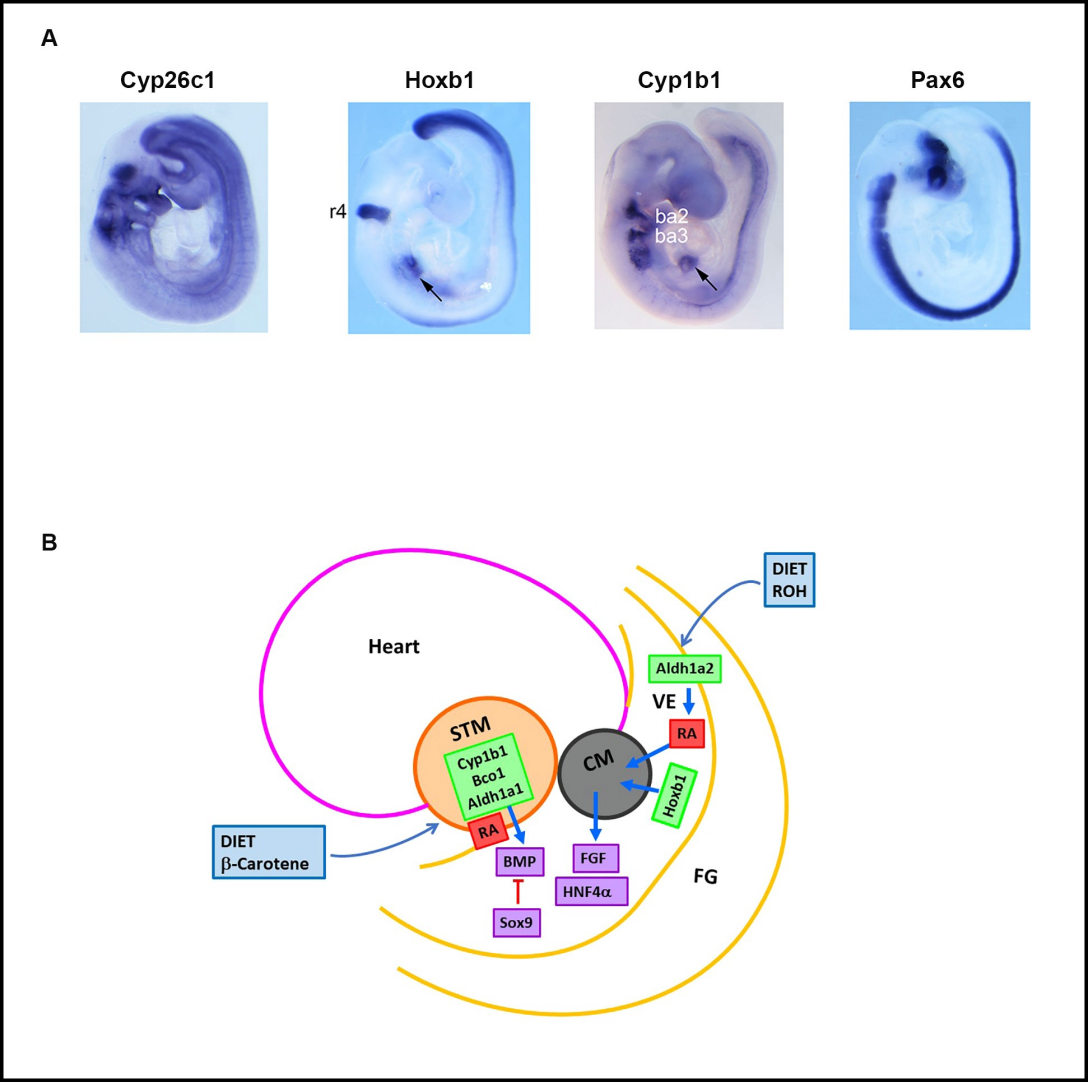
Sites of expression of *Cyp1b1* and *Hoxb1* in WT E9.5 embryos. E9.5 WT embryos from dams administered a vitamin A-sufficient diet were stained by ISH to reveal *Cyp26c1*, *Hoxb1*, *Cyp1b1*, and *Pax6* mRNAs. Lateral views are shown. Noted are hindbrain rhombomere 4 (r4), branchial arches (ba), foregut (ventral endoderm)/septum transverse mesenchyme (STM) (arrow) structures (A). Diagram of foregut (FG) region of liver budding showing positioning of Heart (H), STM and CM (cardiac mesoderm) and ventral endoderm (VE). Sites of expression of *Cyp1b1*, *Bco1* (STM) and *Hoxb1* (VE). Fgf forms are released from CM, and BMP4 from STM, each supporting hepatoblast expansion (Hnf4a). Sox9 marks the posterior region that develops into pancreas (B).

To better address whether Cyp1b1 has an impact on RA activity at E9.5, we examined the expression of RA–associated genes in relation to apparently conserved sites of expression in the hindbrain, branchial arches and foregut **(Fig 5A**, lateral; **S1A Fig**, dorsal). Hoxb1 and Cyp26 forms each have high affinity responses to RA [27, 28, 35, 50]. Pax6 has a more complex dependence on RA but also exhibits regulation of Cyp1b1 [19, 68]. *Hoxb1* has precise location in r4 and in the ventral endoderm of the foregut close to the site of initial liver budding. *Hoxb1* is absent from the three branchial arches but is necessary to initiate the migration of neural crest cells to these sites [69, 70]. A common feature of these regulatory processes that has been extensively studied in the hind brain is the spatial separation of RA synthesis (*Aldh1a2*), metabolism (*Cyp 26* forms) and function (*Hoxb1*).

Classic RA synthesis functions through NAD(P)+ dehydrogenases [43–46], whereas Cyp1b1 in vitro converts retinol and retinaldehyde to RA via NADPH-POR reactions, which have very different metabolic and oxygen requirements [8, 19, 23] that are appreciably shared by the RA-clearance mediated by Cyp26 forms [50]. Cyp1b1 additionally suppresses oxygen stress in vascular and mesenchymal cells [9, 10, 25]. These considerations provide a flexibility to RA homeostasis that we probe here by introducing more extreme metabolic restrictions on the limiting maternal transfer of retinol. This impact of limiting maternal transfer been established at E14.5 [33] but has not previously been examined at E9.5, when previous effects of embryo RA synthesis disruption has been examined [43, 44, 46]. The retinol transfer is particularly critical at this early time [32].

**Fig 6:**
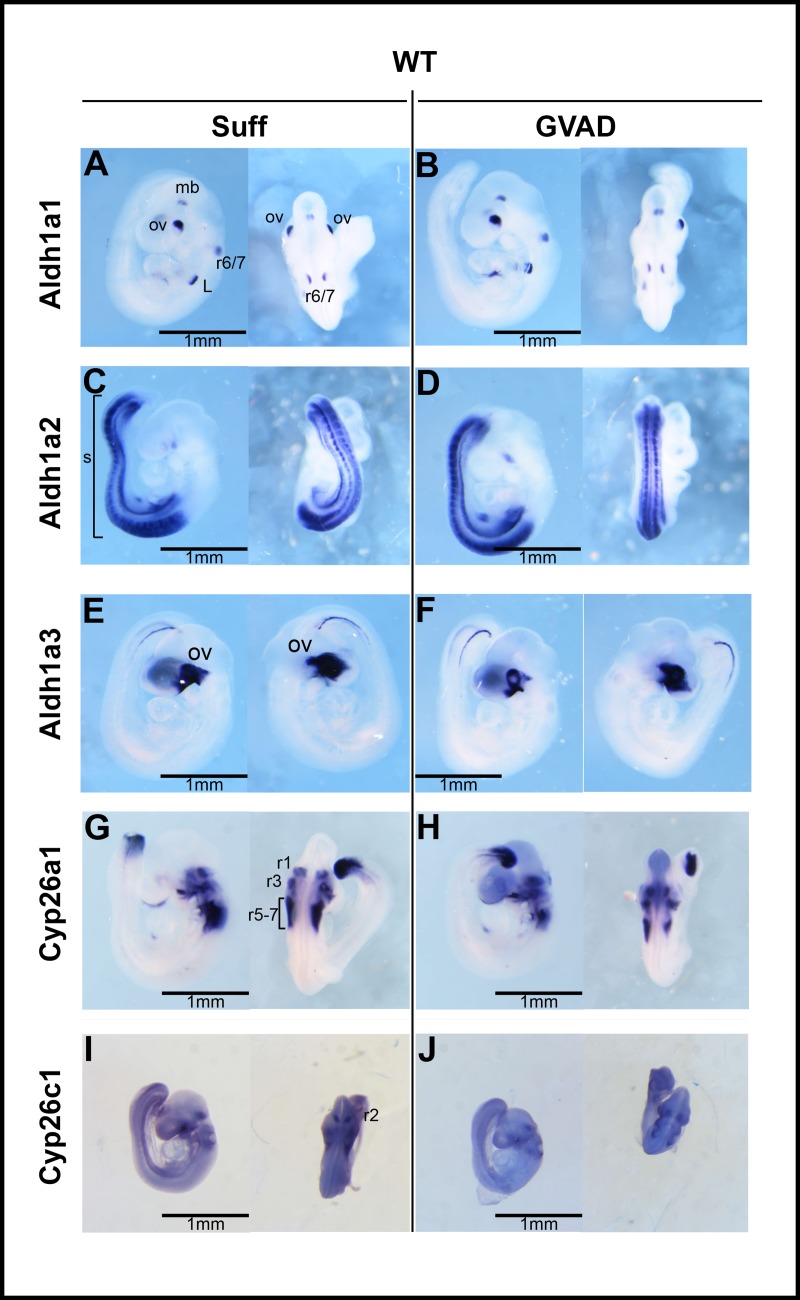
Effect of GVAD on expression of retinoid homeostasis genes. Lateral (left) and dorsal (right) views after whole-mount *in situ* hybridization of E9.5 WT embryos. Dams were administered a vitamin A-sufficient (Suff) or -deficient (GVAD) diet starting at E4.5. Embryos were probed for retinoic acid-generating RNAs: *Aldh1a1*, *Aldh1a2*, and *Aldh1a3*, and retinoic acid-degrading RNAs: *Cyp26a1* and *Cyp26c1*, each as designated. For *Aldh1a3*, left lateral (left) and right lateral (right) views are shown (E, F). Selective expression is noted for *Aldh1a1* in rhombomere (r)6/7, midbrain (mb), foregut/presumptive liver site (A, B); for *Aldh1a2* in somites(s) (C, D); for *Aldh1a3* in optic vesicle (ov) (E, F); *Cyp26a1* in r1, r3, r5, r7 (G, H) and *Cyp26c1* in r2, r4, r6 (I, J). All images are shown to the same scale.

High expression of *Cyp26c1* maintains low RA in r2, r4, r6 and r8, each from the dorsal side, perhaps to minimize effects on the ventral migration of neural crest cells to the branchial arches. There is low dispersed expression in the posterior embryo apart from high expression at the expanding tip. *Cyp26a1* has high complementary expression in r3, r5 and r7 but is essentially absent from the posterior embryo apart from the tip of the tail (**Fig 6G)**.

The separation of *Cyp1b1* from *Hoxb1*, *Pax6* and *Cyp26c1* within r4 is highlighted in the enlarged r4 dorsal views (**S1B Fig**). In r4, the lateral view, dorsal *Hoxb1* is mostly separated from the more ventral *Cyp1b1*. The dorsal view shows that the split in the *Hoxb1* zone produced by the neural groove is absent for Cyp1b1**-/-**. *Pax6*, which does not segregate expression between the different hindbrain rhombomeres (r3-8), is positioned on the dorsal side of *Cyp1b1*. *Pax6* exerts constraints on the rhombomere boundaries [68]. In **S1C Fig**, we have diagrammed the spatial relationship and distribution around the neural groove of the four genes in the r4 region that are shown in **S1B Fig**. *Cyp26c1* and *Cyp1b1* are appreciably separated on either side of *Hoxb1* in r4.

**Fig 5B** shows the spatial relationship between *Cyp1b1* and *Hoxb1* in the foregut region at E9.5 in relation to *Adlh1a2* and *Bco1* (**Figs 4B and 5A**). These liver progenitor cells (hepatoblasts), which are controlled by Hnf4a [11, 42, 64, 71], are jointly activated by Fgf forms from cardiac mesoderm and by BMP4 from the STM [25]. RA can intervene by stimulating the Hoxb1 effects on Fgf forms [35, 41, 42] or by enhancing Bmp4 effects [72]. Potential RA contributions are again spatially and metabolically separated. *Aldh1a2*, which receives retinaldehyde from *Rdh10*, locates close to *Hoxb1* in the foregut endoderm. The STM expresses *Bco1*, which generates retinaldehyde from dietary β-carotene and is co-expressed with *Cyp1b1*, a further potential source of RA. These foregut RA processes are substantially different from those in the hindbrain because of the much lower RA clearance activity provided by near absence of *Cyp26a1* and the relatively low *Cyp26c1* presence.

**Fig 7:**
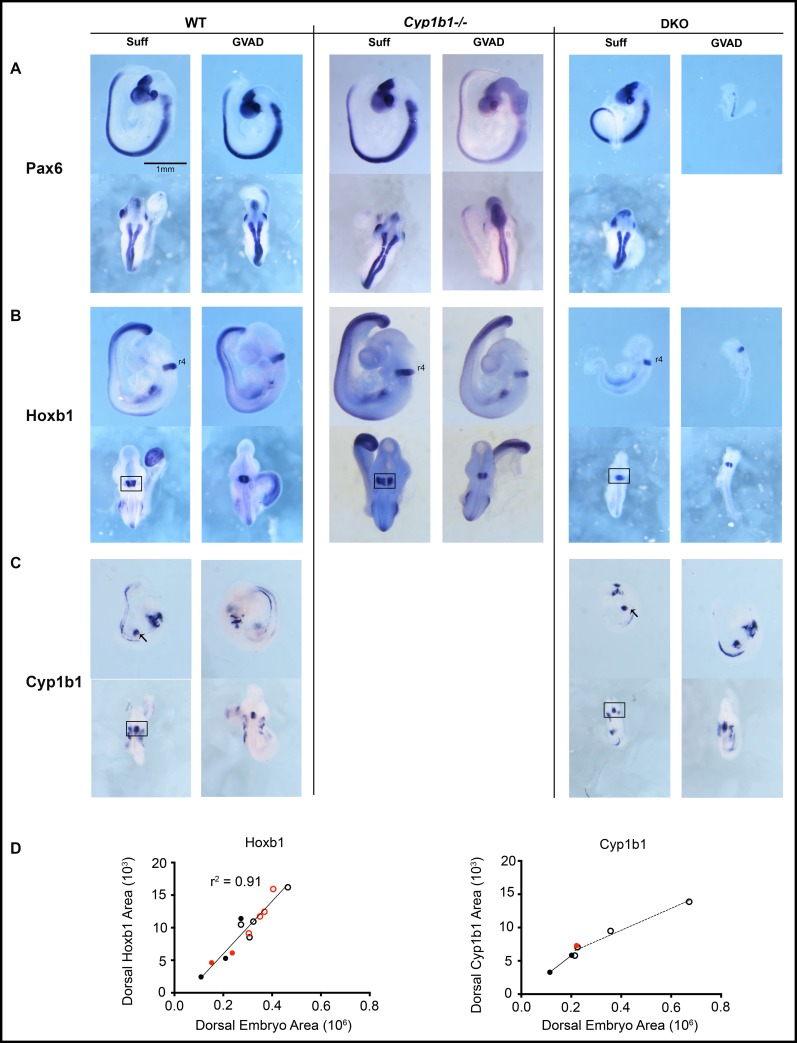
Effects of GVAD on morphogenic gene expression patterns in WT, Cyp1b1-/-, and DKO embryos. E9.5 WT (left), Cyp1b1-/- (middle), and DKO (right) embryos from dams administered vitamin A-sufficient (Suff) or -deficient (GVAD) diets are shown. DKO embryos are compared to WT embryos in a similar size range (Fig 2A). Embryos were probed by ISH to evaluate expression patterns of *Pax6* (A), *Hoxb1* (B), and *Cyp1b1* (C). Lateral (upper) and dorsal (lower) views are provided. GVAD DKO embryos were very variable in size. A third were similar to Suff DKO, while the remainder were smaller and often malformed. All images are shown to the same scale. Analysis of r4 absolute expression areas of *Hoxb1* and *Cyp1b1* each from a dorsal view of ISH images from WT (open circles) and DKO embryos (solid circles) from Suff (black) or GVAD (red) diets (D). For ISH images see S4 Fig.

In addition to the hindbrain and foregut, *Hoxb1* is expressed close to *Cyp1b1* in the somites (**Fig 7B**), again adjacent to *Aldh1a2* as a prime source of RA generation. *Aldh1a2* also overlaps with *Cyp1b1 and Hoxb1* in the somites (**Fig 6C**).

### 3.7 Maternal GVAD does not alter distribution of retinoid metabolism genes at E9.5

Local concentrations of RA are determined by the balance between Aldh1a-mediated retinaldehyde dehydrogenase activity and RA removal by Cyp26-mediated 4-hydroxylation. At E14.5, the GVAD diet removes all retinyl esters from the dams and about half of the esters from the embryos (**Fig 3B**) but has no effect on any of the Aldh1a forms. Aldh1a2 is the main form in the embryo [47]. Aldh1a3 is specific to the optic vesicle (**Fig 6E and 6F**).

Aldh1a1, which is the major source of RA generation in the liver, has specific expression sites, presumably to provide local RA. Aldh1a1 is expressed adjacent to Cyp1b1 both in the brain (r6/7) and in the foregut [44] and is, therefore, a source of RA at these sites.

These two Cyp26 forms effectively metabolize RA but are also highly dependent on transcriptional stimulation by RA, thereby providing homeostatic control [73]. *Cyp26a1* is expressed in alternate rhombomeres (r1, r3, r5 and r7) with *Cyp26c1* (r2, r4, and r6) (**Fig 6G–6J)**. *Cyp26a1* has minimal expression outside the hindbrain apart from the posterior tip. *Cyp26c1* has broader expression, particularly at sites close to *Cyp1b1*, including r4, the foregut and somites. Cyp26b1, which is not probed here, is the main form in the liver at birth **(Table 1**). GVAD at E9.5 also has no effect on Cyp26a1 and Cyp26c1.

### 3.8 Absence of an effect of Cyp1b1 deletion and GVAD diet on *Hoxb1* and *Pax6* expression at E9.5

**Fig 7A–7C** shows the impact of lowering dietary retinol through GVAD and DKO manipulations on the expression patterns. We show expression of *Cyp1b1* and two RA-responsive genes, *Hoxb1* and *Pax6*. *Cyp1b1* sustains remarkably constant expression ratios for the various sites (hindbrain (r4), branchial arches, foregut and somites) for size matched WT and DKO embryos, irrespective of GVAD treatment (**Figs 4A, 7C** and **S3A**). The positioning of *Hoxb1* on the dorsal side of r4 and spatially extended *Pax6* expression function as precise markers of E9.5 staging. The larger Suff DKO embryo shows more extensive expression of *Pax6*, indicative of full E9.5 staging.

Neither Cyp1b1 deletion with the Suff diet nor application of the GVAD protocol to either WT or Cyp1b1-/- dams impacts *Hoxb1* or *Pax6* in the hindbrain. A consistent *Pax6* pattern is also seen, irrespective of appreciable differences in embryo size (**Fig 7A and 7B and S2 Fig)**. For *Hoxb1*, the key sites of expression in the foregut and the somites are similarly retained. The size variation of these embryos occurs with retention of patterning for *Aldh1a2*, *Pax6* and *Hoxb1* (**S2 Fig**). Again, there is no effect of Cyp1b1 deletion or the GVAD deficiency on either this patterning or the embryo size.

The dorsal r4 images for *Hoxb1* and *Cyp1b1* provide specific areas to link their relative expressions as a function of growth or projected dorsal body area (**S4 Fig**). For WT and DKO embryos with Suff and GVAD treatments, this r4 *Hoxb1* area decreased 2.5-fold in direct proportion to the embryo size across all treatments (r^2^ = 0.9) (**Fig 7D**). *Cyp1b1* expression declines much less for the same treatment groups, although with steeper decline for the DKO embryos. The *Cyp1b1* in r4, branchial arches, and foregut/STM occupies a larger proportion of the embryo for small embryos than for large embryos, irrespective of treatment (**S3** and **S4** Figs). In contrast to the chick model, which is reported to be RA-dependent [13], mouse Cyp1b1 has distal Hox and Pax6 elements, but no evidence for direct RA dependence [19].

### 3.9 DKO embryos show site selective effects on Hoxb1 suppression

The drastic decrease in retinol supply to DKO embryos at E9.5 produces a size range that overlaps with the smaller WT embryos, although the average size is smaller (**Fig 3C**). In the Suff DKO embryos, *Hoxb1* is sustained in r4, but lost in somites and the ventral foregut endoderm, the site of liver budding (**Figs 7B** and **S3B**). The pair of GVAD DKO embryos completely loses foregut *Hoxb1* expression, even though they are larger than the Suff DKO.

GVAD also interacts with the DKO genotype to decrease lateral forebrain evagination, a characteristic feature of E9.5 embryos (n = 114) (DKO 71% Suff, 87% GVAD; WT 19% Suff, 27% GVAD) [74].

### 3.10 DKO embryos adapt with lower hindbrain *Cyp26a1*, but increased foregut *Bco1*

**Fig 8:**
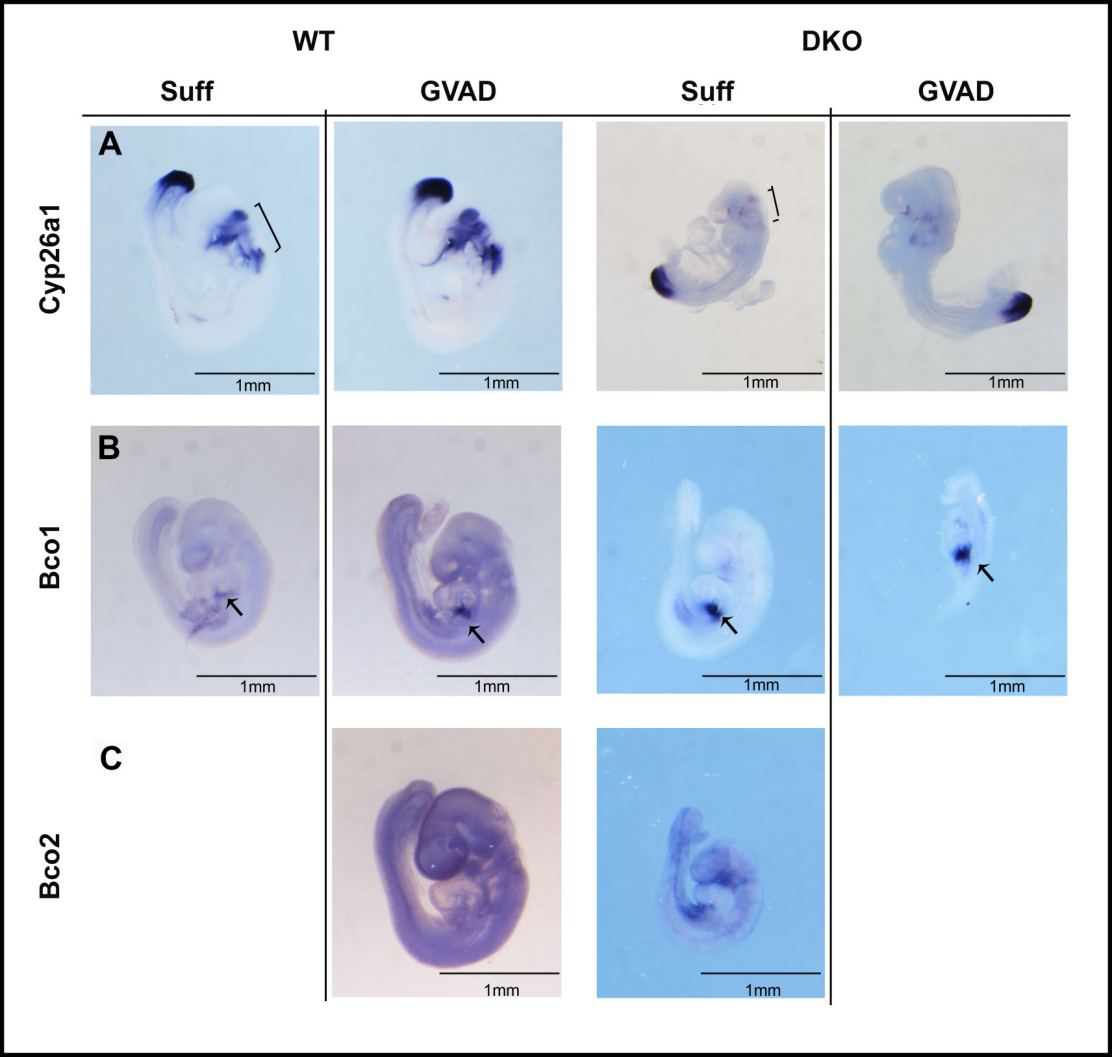
Effect of DKO and GVAD on E9.5 whole-mount ISH expression of genes associated with retinoid metabolism. E9.5 WT or DKO embryos from dams administered Suff or GVAD diets were probed by ISH for *Cyp26a1* (A), and β-carotene metabolizing genes: *Bco1* (B) and *Bco2* (C). All images are shown to the same scale. For each, only the lateral view is shown. Hindbrain (bracket) and foregut presumptive liver (arrow) structures are noted. *Bco1* staining was much stronger than *Bco2*.

Decreased expression of *Cyp26a1* and *Cyp26c1* can accommodate a large decrease in RA transfer into the hindbrain gradient due to the diminished transplacental retinol transfer for GVAD DKO embryos [33]. Removal of posterior synthesis genes (Aldh1a2 and Rdh10 deletion) produces similar hindbrain effects from decreased posterior RA synthesis [44]. *Cyp26a1*, which is potently induced by RA [73], is almost completely depleted from r3 and r5 in DKO embryos with either Suff or GVAD diets, even though expression in the posterior site of somite expansion is unaffected (**Fig 8A**). A net decrease in *Cyp26a1* expression in GVAD DKO embryos is also shown by qPCR at E14.5 [33]. This extensive hindbrain decrease in *Cyp26a1* induction is consistent with a decline in hindbrain RA. This lowering of RA metabolism appears to be sufficient to maintain the normal RA gradient to r4 and the associated morphogenesis. This loss of *Cyp26a1* places these embryos close to the limit of RA homeostasis adjustment. The morphological changes in some DKO embryos, including the delayed forebrain evagination, particularly on the GVAD diet, support this perspective.

*Bco1*, which can supply retinaldehyde to the foregut region [60], is enhanced in DKO embryos, including in a normal embryo from the GVAD diet (**Fig 8B**, **S3 Fig**). The low expression of the mitochondrial form, *Bco2*, also increases in the ventral foregut (**Fig 8C**). The robust expression of *Bco1* provides a means to accommodate diminished vitamin A/retinol through metabolism of dietary β-carotene [75].

## 4. Discussion

### 4.1 Cyp1b1–deficiency and retinol deficiency in E9.5 embryos

This research started from the finding that expression of mouse Cyp1b1 in an RA-deficient quail development model generated specific effects of RA [13]. Others studying the families of genes that metabolize retinol and retinaldehyde (Aldhs, Radhs, Raldhs) had also found that deletions of the major genes left residual RA activity in the hindbrain at mid-gestation (E9.5). A double deletion of Raldh10 and Cyp1b1 showed that Cyp1b1 did not account for this residual activity [44]. More extensive probing presented here establishes that *Hoxb1*, which is selectively sensitive to RA, is unaffected by deletion of *Cyp1b1*, even in combination with severe depletion of retinol. During pregnancy in mice, this deficiency (GVAD) only starts to produce retinol deficiency in the late fetal stage. We show that at E9.5, *Hoxb1* and *Pax6*, another RA-sensitive gene, retain their E9.5 patterning in surviving mice with *Lrat/Rbp4* double deletions (DKO) [33]. However, *Cyp1b1* expression in this model is highly persistent, retaining all sites of expression. This leaves the question of the function of this conserved *Cyp1b1* expression.

### 4.2 Cyp1b1 and dietary retinol selectively support postnatal increases in gene expression and also stellate and *Hamp* changes at birth

At birth, GVAD treatment lowers liver retinoids, severely depleting retinol in the postnatal period **(Fig 2C and 2D)**. The Cyp1b1-/- genotype and retinol depletion indeed show overlapping effects on many genes that increase in response to the postnatal rise in Igf1 [76]. Particularly affected are those previously linked to either *Srebp-1c* (9 oleate pathway genes) or *Srebp2* (12 cholesterol pathway genes) [4]. These *Srebp-1c*-regulated genes are among only 30 that are suppressed in WT mice after GVAD. These *Srebp-2*-regulated genes are among about 50 genes for which the postnatal increase depends on *Cyp1b1*, but not retinol. Instead, GVAD fully restores WT Srebp2 activity to *Cyp1b1-/-* pups. Thus, about 80 of these 500 increases depend almost completely on Cyp1b1, but with distinct modes of retinol participation typical of either *Srebp-1c* or *Srebp2* regulation [77,78] that encompass both cholesterol and retinoid homeostasis [79,80]

Earlier, at birth, liver gene expression shows only two effects of GVAD, but again, each is shared with Cyp1b1 deletion: stimulation of the markers of stellate cell activation [14] (**Table 2, Fig 2E**) and suppression of the iron regulatory gene, *Hamp*. Srebp 1c contributes to *Hamp* expression [81]. Activation of stellate cells is also integrated with Srebp regulation [82, 83]. Srebp regulation may in some way connection to Cyp1b1 and retinol. Although *Hamp* retains this connection up to weaning, Srebp regulation of lipogneic genes is seen only for the postnatal stimulation.

### 4.3 Do perinatal effects of Cyp1b1 deletion arise from expression earlier in development?

The liver is targeted extensively by Cyp1b1, even though *expression* is scarcely detectable. Earlier expression of *Cyp1b1* in the developing liver provides one source for disruption of participation, since this occurs close to sites of RA synthesis in the foregut site of liver initiation. However, GVAD-and Cyp1b1-/- changes do not affect the patterning of RA-responsive genes, including *Hoxb1* [35] (**Fig 7B and 7C**). High local expression of *Cyp26* forms *a1* and *c1* adjust RA deficiencies and can obscure removal of Cyp1b1 contributions, since complete deletion of either form is insufficient to affect RA activity [50, 84]. Suppressed maternal retinoid transfer in the *Lrat*/*Rbp4* DKO model is sufficient to remove hindbrain *Cyp26a1* and probably RA-dependent *Cyp26c1*. RA selectivity is such that residual RA is sufficient to sustain *Hoxb1* expression (**Fig 8**), which correlates closely with embryo size (r^2^ = 0.9) (**Fig 7D**). *Cyp1b1* expression is, however, sustained at all sites (**Fig 7D**). With near absence of countering Cyp26 metabolism, a low Cyp1b1 contribution may be sufficient to maintain the RA-sensitive Hoxb1 [35].

*Cyp1b1* has notably strong expression in the STM, which supports liver budding [41, 42]. This expression is located close to *Bco1*, an oxygenase that generates retinaldehyde from β-carotene. *Bco1* is particularly evident in DKO embryos. Aldh1a1, which is also present, and Cyp1b1 provide alternative conversions of retinaldehyde to RA that depend on, respectively, NADP+ and NADPH (**Fig 5**).

Liver budding requires a partnership between Bmp4 from the STM and Fgf forms from the cardiac mesoderm [41–43]. At E9.5, hepatoblasts, sustained by Hnf4a, expand with proliferative stimuli provided by Fgf, with support from RA via Aldh1a2 and dietary retinol. Penetration into the STM is promoted by Bmp4, which is commonly supported by RA. Here, Bco1 and, potentially, Cyp1b1 may deliver this support. STM also provides the source of stellate cells that exhibit dual retinol–Cyp1b1 regulation in the liver at birth [38].

### 4.4 A systems approach to GVAD-Cyp1b1 regulation

**Fig 9:**
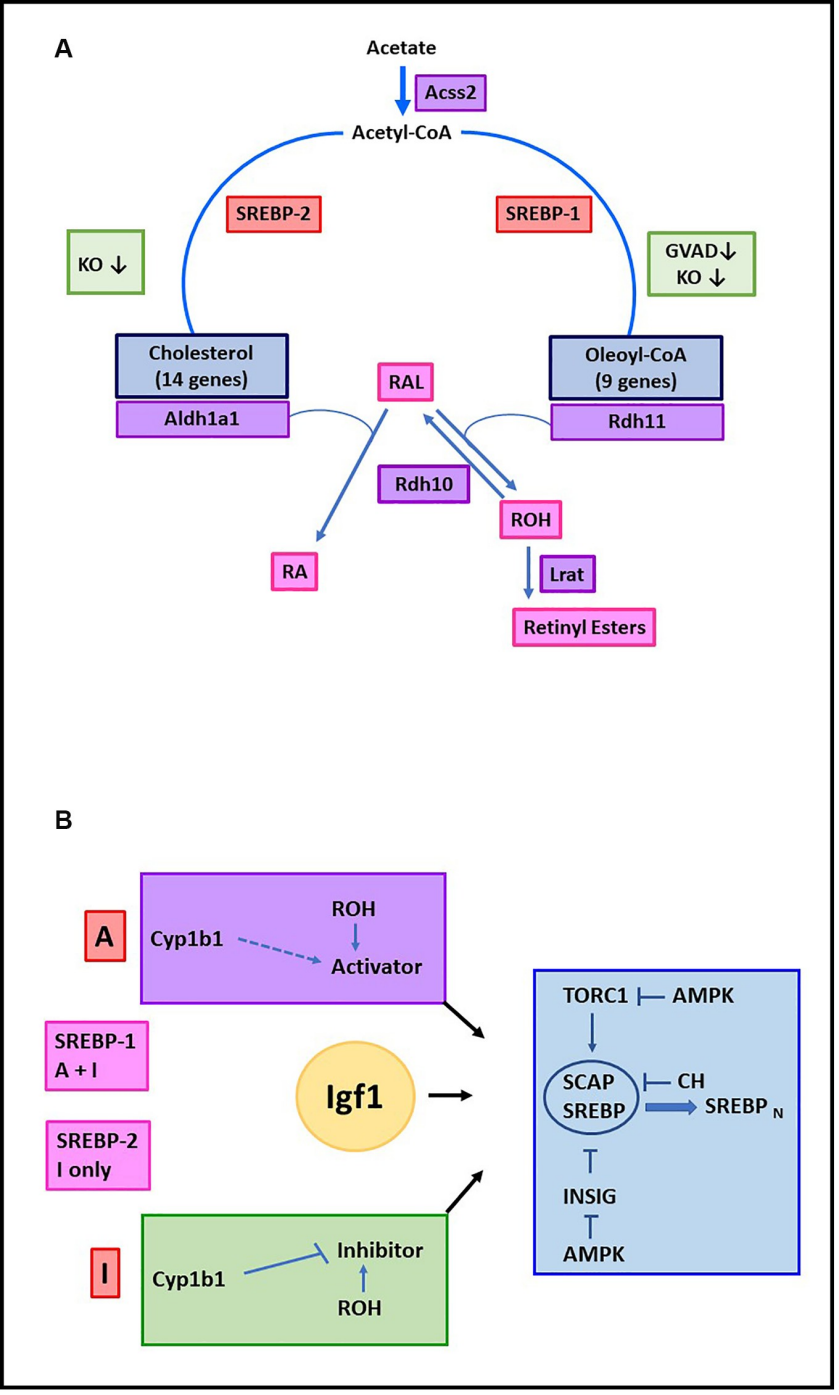
Dual Cyp1b1/retinol regulation of Srebp-1c/oleate and Srebp2/cholesterol pathways in postnatal liver. Pathways that convert acetate to cholesterol or oleoyl-CoA and retinaldehyde to RA or retinyl esters. All processes are inhibited in Cyp1b1-/- pups (KO) (Table 1). Srebp2/cholesterol pathway genes and Aldh1a1 show no effect of GVAD in WT pups. The Srebp-1/oleoyl-CoA pathway genes show losses with GVAD treatment of WT pups. The retinaldehyde is formed in the liver from β-carotene by Bco1. The reductase, Rdh11, which regenerates retinol, follows the Srebp1 pattern. This step, followed by Lrat, generates retinyl esters in stellate cells. Aldh1a1 is the major means to convert retininaldehyde to RA (A). Distinctive Cyp1b1 –retinol partnerships for Srebp1 and Srebp2 during stimulations by Igf1. Inhibition Pathway (I): Cyp1b1 removes/metabolizes the mediator of an inhibitory pathway, which becomes active in Cyp1b1-/- pups. Retinol supports this inhibitory pathway, leading to stimulation with GVAD in Cyp1b1-/- pups, but no effect in WT, where Cyp1b1 removes the mediator. Activator Pathway (A): Retinol stimulates an Activator Pathway that leads to the suppression by GVAD in WT pups. A contribution of Cyp1b1 to activation is possible, but Cyp1b1-/- Activator losses cannot be distinguished from enhancement of the Inhibitor Pathway. TORC1 activation and Insig inhibition control the Scap-Srebp complex that is suppressed by cholesterol (CH) (B).

Key differences between Srebp-1c and Srebp-2, with respect to retinol participation, have been identified. Genes regulated by Srebp-1c show suppression by GVAD in the WT litters, while genes regulated by Srebp 2 resist this effect. Signaling by each transcription factor is largely removed in Cyp1b1-/- pups but is reversed to WT levels by GVAD. **Fig 9** shows pathways that are linked to these factors (A) and a network model to account for Srebp-1/Srebp-2 differences (B). Additional genes show expression signatures typical of the Srebp-1 (*Lpin1*, *Gaddd45g*) or Srebp2 *(Insig1*, *Thrsp2*). These signatures have diverse roles in lipogenic processes [85–89], but Srebp participation remains to be determined for many of these genes.

Postnatal gain in body weight in WT pups increases in proportion to activation of Srebp-1c genes, but not in Cyp1b1-/- pups or for Srebp2 genes (**Fig 2**). This selectivity points to important links to energy regulation. Srebp forms are retained in the endoplasmic reticulum (ER) by a complex with the cholesterol sensor, Scap. This complex transfers to the Golgi for cleavage to the active nuclear form when cholesterol becomes depleted [90]. Srebp activation is also stimulated via the multi-protein mTORC1 complex, a sensor of metabolic homeostasis [76, 91, 92]. mTORC1 transmits insulin and Igf1 signaling to phosphorylation of Srebp, subject to the various sensor modulations [93]. Insig enhances cholesterol-dependent resistance to Scap-Srebp activation, which is also removed by insulin or Igf1. AMPK, which is activated when ATP declines, inhibits both Srebp activation processes and connects Srebp regulation to systemic signaling that appears to be Cyp1b1 sensitive [85, 91, 94]. Thus, Cyp1b1 metabolizes estradiol, and many features of the adult Cyp1b1-/- phenotype parallel increased effects of estradiol and changes in the hypothalamus [2]. Estradiol enhancement of leptin signaling, transmitted through the sympathetic nervous system, elevates inhibitory liver AMPK [95–97].

### 4.5 Conclusions and Key questions

Srebp-supported gene expression is comprehensively suppressed in perinatal Cyp1b1-/- pups. Srebp1 and Srebp2 mRNA are unaffected, indicating that their post-translational regulation is targeted. These same genes are substantially affected by a retinol-depleted maternal diet (GVAD) that lowers liver retinol. At birth, GVAD and Cyp1b1 deletion each suppress the iron regulator, *Hamp*, while also increasing multiple stellate activation markers, each a likely target of Srebp control [82, 83]. The low presence of *Lrat* and retinyl esters and enrichment of activated stellate markers suggests an altered basal state that may be more sensitive to these changes [14]. Postnatal increases in Igf1 expression [98] parallel the major increases in hepatocyte gene expression, including the 15 percent that depend on Cyp1b1 and retinol, which include the Srebp1-stimulated lipogenic genes. The links to synthesis of fatty acids, cholesterol and retinoids emphasizes their integrated homeostasis [79] (**Fig 9A**). The control of Scap-Srebp complexes, through mTORC1, Insig and AMPK provide multiple ways for interventions by Cyp1b1 and retinol (**Fig 9B**).

Cyp1b1 is not expressed in perinatal or adult hepatocytes, thus requiring indirect activity through metabolism at remote sites connected through developmental programs or systemic signaling. We show that Cyp1b1 appears at sites of mid-gestation liver budding. Cyp1b1 deletion and GVAD manipulations of pregnancy have no effect on RA-sensitive *Hoxb1* at this site or in the hindbrain. Nevertheless, co-expression of *Cyp1b1* with *Bco1* and *Aldh1a1* in foregut STM, provide possibilities for a Cyp1b1-retinol connection to stellate cells and postnatal Srebp regulation.

Cyp1b1 has multiple substrates and, therefore, several ways to partner retinol [9, 13, 20, 22]. However, delivery of retinoids from the mother and protective Cyp26 metabolism may obscure limiting Cyp1b1 activity. The DKO model minimizes the difficulties of surplus dietary retinol and the buffering effects of Cyp26. In near absence of *Cyp26a1*, *Cyp1b1* expression and RA-dependent *Hoxb1* are conserved in proximity, suggesting conditions for testing their relationship, including assessing local retinoid levels. These DKO embryos survive to adult life [33] and, therefore, the impact of these interventions on neonatal liver adaptations in relation to adult Srebp regulation can be tested. The alternative intervention of Cyp1b1 in systemic inhibitory signaling to Srebp (Fig 9B) is also being tested.

## Highlights

- Multiple stellate activation markers are equally stimulated at birth by retinol deprivation and Cyp1b1 deletion.
- Postnatal liver pathways mediated by Srebp forms depend on Cyp1b1 and retinol; extend to Aldh1a1 and Rdh11.
- Cyp1b1 deletion, even with dietary retinol depletion, does not decrease RA-dependent processes in E9.5 embryos.
- *Lrat/Rbp4* deletions (DKO) remove hindbrain *Cyp26a1*, but retain *Hoxb1* and *Cyp1b1*, including in the foregut.
- During foregut liver budding, associated mesenchyme (STM) co-expresses *Cyp1b1* and *Bco1*, a stellate marker that forms retinaldehyde.

## Summary

Removal of Cyp1b1 or liver retinoids stimulates perinatal stellate activation, with subsequent losses in Srebp-mediated transcription involved in oleate, cholesterol and retinoic acid synthesis. Earlier at E9.5, exceptional lowering of dietary retinol by deletions of both *Lrat and Rbp4* (DKO) removes *Cyp26a1*, which metabolizes RA. *Cyp1b1* and *Hoxb1* retain adjacent expression in the foregut that is sufficient for normal liver initiation. Co-expression of *Cyp1b1* and *Bco1* in local mesenchyme suggests cooperation in RA synthesis that supports BMP4 effects on liver budding, but also regulates stellate cells seen at birth.

## Supporting information

S1 Table Number of embryos used for each ISH probe. (DOCX)

S1 Fig Expression of *Cyp1b1* in the hindbrain relative to morphogenic genes in WT E9.5 embryos. E9.5 WT embryos from dams administered a vitamin A sufficient diet were stained by ISH to reveal *Cyp26c1*, *Hoxb1*, *Cyp1b1*, and *Pax6* mRNAs. Dorsal (A) views are shown. Magnified views of r4 (dotted boxes) are shown (B). Relative distributions of *Cyp26c1*, *Hoxb1*, *Cyp1b1*, and *Pax6* in r4 adjacent to the neural groove (NG) are diagrammed (C).
(TIF)

S2 Fig Somite number that determines developmental stage displays a range in embryo size. Lateral views of *Aldh1a2* (A), *Pax6* (B), and *Hoxb1* (C) staining demonstrate a range of RNA domain sizes in E9.5 (21–29 somites) WT Suff embryos. All images are shown to the same scale.
(TIF)

S3 Fig Lateral views of *Hoxb1*, *Pax6*, *Bco1*, *and Bco2* expression in WT and DKO embryos in relation to *Cyp1b1* and the impact of GVAD. *Cyp1b1* (A), *Hoxb1* (B), *Pax6* (C), *Bco1* (D) and *Bco2* (E) ISH staining of multiple DKO embryos from dams on Suff and GVAD diets are compared to a representative WT embryo from a dam on a Suff diet. Embryo sizes are proportional to their projected cross sectional areas (Fig 2). All images are the same scale.
(TIF)

S4 Fig GVAD effect on *Hoxb1* and *Cyp1b1* r4 expression in WT and DKO embryos. Dorsal views of WT (A and C) and DKO (B and D) embryos from dams on Suff (left) and GVAD (right) diets are compared.
(TIF)

## Abbreviations

Cyp1b1: cytochrome P450 1b1
RA: retinoic acid
GVAD: protocol consisting of maternal early-gestational initiation of a VA-deficient diet
STM: septum transversum mesenchyme
POR: NADPH P450 oxidoreductase
DKO: *Lrat-/-Rbp-/-*
WT: wild type, C57Bl/6J mice
E: embryonic day
Suff: indicating administration of a VA-sufficient diet
ISH: *in situ* hybridization
NC: neural crest
Ba: branchial arch
