# Supplementary material for: Cyp1b1 directs Srebp-mediated cholesterol and retinoid synthesis in perinatal liver; Association with retinoic acid activity during fetal development
Source: PLoS One. 2020 Feb 6;15(2):e0228436. doi: 10.1371/journal.pone.0228436 (PMC7004353; doi:10.1371/journal.pone.0228436)
Supplement: S1 Table — (DOCX) [file pone.0228436.s001.docx]

**Supplementary Table 1**. Number of embryos used for each ISH probe.

|  | WT | | Cyp1b1-/- | | DKO | |
| --- | --- | --- | --- | --- | --- | --- |
| **ISH Probe** | Suff | GVAD | Suff | GVAD | Suff | GVAD |
| *Cyp1b1* | 11 | 11 | - | - | 3 | 2 |
| *Hoxb1* | 7 | 7 | 4 | 4 | 4 | 2 |
| *Pax6* | 7 | 8 | 4 | 3 | 2 | 1 |
| *Cyp26a1* | 6 | 6 | - | - | 2 | 1 |
| *Cyp26c1* | 7 | 8 | - | - | - | - |
| *Adh1a1* | 3 | 5 | - | - | 3 | 2 |
| *Aldh1a2* | 7 | 9 | 2 | 2 | - | - |
| *Aldh1a3* | 3 | 5 | 2 | 1 | - | - |
| *Bco1* | 4 | 4 | - | - | 3 | 2 |
| *Bco2* | 4 | 4 | - | - | 3 | 2 |
|  |  |  |  |  |  |  |
| Total | 59 | 67 | 12 | 10 | 20 | 12 |
