# Supplementary figures and images for: Cyp1b1 directs Srebp-mediated cholesterol and retinoid synthesis in perinatal liver; Association with retinoic acid activity during fetal development
Source: PLoS One. 2020 Feb 6;15(2):e0228436. doi: 10.1371/journal.pone.0228436 (PMC7004353; doi:10.1371/journal.pone.0228436)

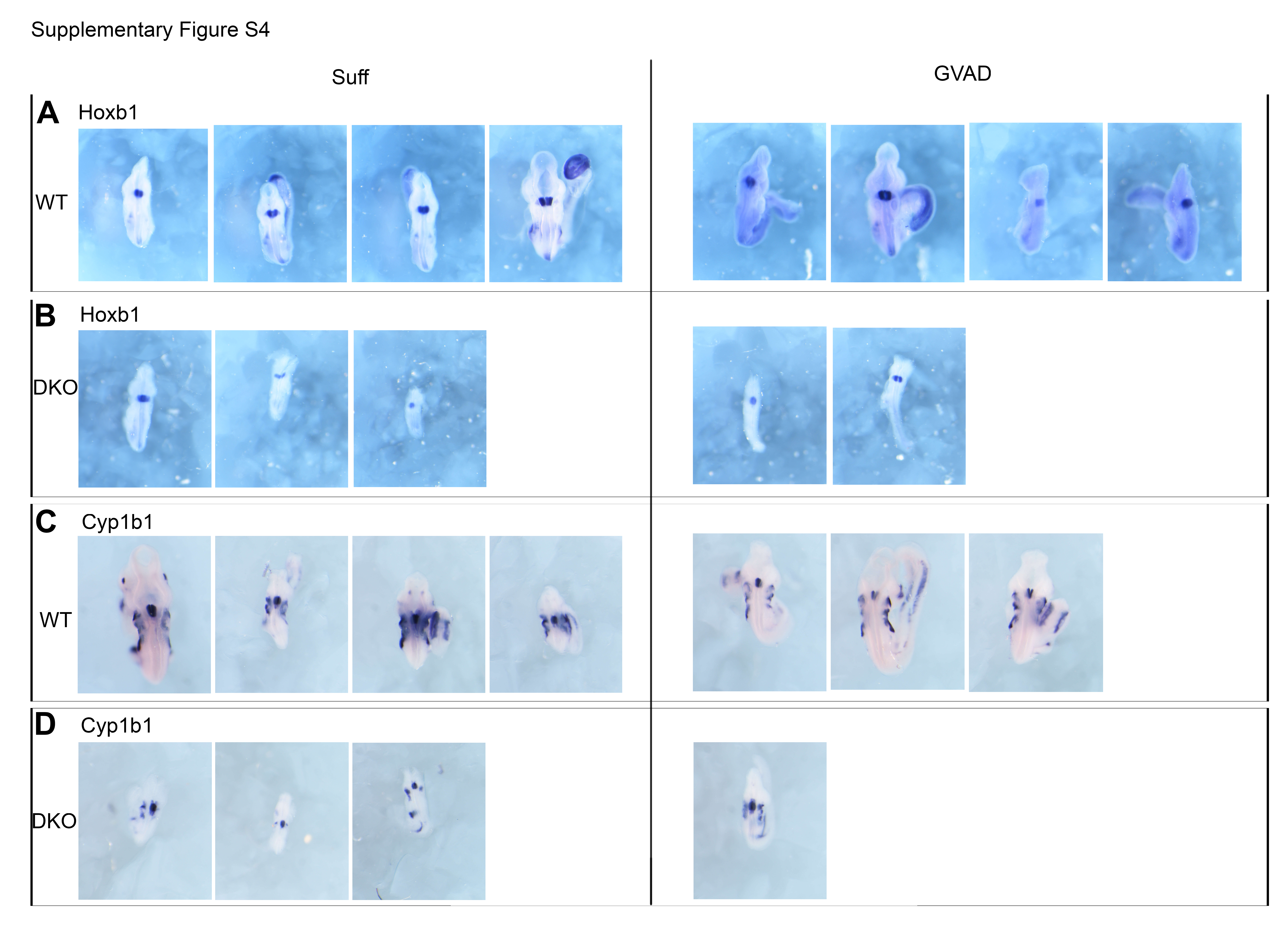

Supplement: S4 Fig — Dorsal views of WT (A and C) and DKO (B and D) embryos from dams on Suff (left) and GVAD (right) diets are compared. (TIF) [file pone.0228436.s005.tif]
